# Phylogeny of Merlin’s grass (Isoetaceae): revealing an “*Amborella* syndrome” and the importance of geographic distribution for understanding current and historical diversity

**DOI:** 10.1186/s12862-022-01988-w

**Published:** 2022-03-16

**Authors:** Eva Larsén, Niklas Wikström, Anbar Khodabandeh, Catarina Rydin

**Affiliations:** 1 Department of Ecology, Environment and Plant Sciences, Stockholm University, 106 91 Stockholm, Sweden; 2 Bergius Foundation, The Royal Academy of Sciences, Box 50005, 104 05 Stockholm, Sweden

**Keywords:** Eastern Cape, *Isoetes*, *Isoetes wormaldii*, Phylogeny, Species distribution, Dispersal, Speciation, Polyploidy

## Abstract

**Background:**

Merlin’s grass (*Isoetes*, Isoetaceae, Lycopsida), is the extant remnant of the isoetalean wood-producing lycopsids that originated during the Paleozoic, possibly in aquatic or boggy habitats. Modern day species are aquatic, semi-aquatic or terrestrial and occur almost worldwide. They display little morphological variation; the lobed corm has helically arranged leaves with internal air channels and basal sporangia. Genetic variation has also proven limited, which has hampered phylogenetic inference. We investigate evolutionary relationships in *Isoetes*, using molecular data and an extended sample of species compared to previous work, adding species that have never before been included in a phylogenetic study.

**Results:**

Our results reveal an unexpected discovery of an “*Amborella* syndrome” in Isoetaceae: a single poorly known species is sister to the remaining family. The species, *Isoetes wormaldii*, is a rare endemic to the Eastern Cape of South Africa. Its leaves are flattened with a rounded point, which sharply contrasts with the awl-shaped leaves of most other species of *Isoetes*. The remaining species of *Isoetes* are resolved in five major clades, also indicated in previous work. While the phylogeny shows geographic structure, the patterns are complex. For example, tropical-southern African species occur in at least five clades, and Indian, Australian and Mediterranean species in at least three clades each.

**Conclusion:**

The evolutionary and biogeographical history of *Isoetes* is not easily explained, and may conceivably include ample extinction and a mixture of ancient and more recent processes. Previously shown difficulties with node age estimation increase the problem. The here demonstrated sister-relationship between the phylogenetically, morphologically and genetically distinct *Isoetes wormaldii* and the remaining family appears to bridge the morphological gap between *Isoetes* and its extinct relatives, although further studies are needed. Moreover, it shortens the branch length to its living sister genus *Selaginella*, and may enhance node age estimation in future studies. *Isoetes wormaldii* is critically endangered, known only from one (to a few) minor populations. Immediate actions need to be taken if we want to prevent this unique species from going extinct.

**Supplementary Information:**

The online version contains supplementary material available at 10.1186/s12862-022-01988-w.

## Background

Merlin’s grass, *Isoetes* of the Lycopsida, are lone survivors of a once much more diverse group of wood-producing lycopsids that also included the Paleozoic tree-lycopods (the rhizomorphic lycopsids sensu DiMichele and Bateman 1996, [1]). The extant genus *Isoetes* (Isoetaceae) has a nearly worldwide distribution [2–5] but fairly restricted habitat preferences in that most species live semi-aquatically in habitats that are seasonally inundated. There are purely aquatic and terrestrial species as well [2–6], and repeated transitions between aquatic/semi-aquatic and terrestrial habitats have been inferred for the genus [7, 8]. *Isoetes* plants are generally quite small but some species may reach a height over 50 cm or more [4, 5]. The stem takes the form of a lobed corm [3–6], to which the leaves are helically attached forming a basal rosette [4, 5]. The leaves usually have four air channels [4, 6] (although exceptions have been reported, [9]) and may have mega- or microsporangia sunken into the base of the leaf. The sporangia are often wholly or partly covered by a thin tissue called velum, and a ligule is attached to the adaxial side of the leaf distal to the sporangia [4–6]. The presence of a ligule (a small often triangular extension of tissue on the adaxial side of the leaf, [4]), as well as heterospory, are characteristics that Isoetaceae share with their closest living relatives, the Selaginellaceae [10, 11, 12, 13], as well as with their extinct relatives in the remaining Isoetales (the rhizomorphic lycopsids), a lineage that can be traced at least to the Late Devonian [1, 10, 11, 14, 15, 16].

### Speciation, species delimitation, ploidy levels

There are at least 200 extant species of *Isoetes* [3, 17] that vary relatively little in general morphology despite their widely dispersed localities. Many are rare with restricted geographic distributions [2–4, 6] but there are also species that are widespread over large areas (at least as currently circumscribed). Examples of the latter are the largely circumboreal *Isoetes lacustris* and *I. echinospora*. Evolution of morphological characters in the extant Isoetales (i.e., the genus *Isoetes*) has been investigated but has often been considered problematic to assess as *Isoetes* displays an unusual combination of morphological stasis yet with high variation and ample parallel evolution within that conserved framework [18]. Species delimitations in *Isoetes* are typically not questioned, but species of the genus are considered difficult to identify [3, 8, 19] and recent work on Mediterranean species [20] indicate that taxonomic problems may have been overlooked, in particular for non-North American species. Further, polyploidy and hybridization are frequent in the genus [e.g., 3, 8, 21, 22, reported to result in sterile as well as fertile individuals. For several species, multiple ploidy levels within species are reported (see e.g., summaries in Refs. [23, 24]).

### Dispersal and reproduction

There have been discussions about how the spores of *Isoetes* are dispersed [5, 8, 23, 25, 26]. Troìa [24] summarized state-of-the-art of the topic and found that much of the information appears largely anecdotal. The heterosporous condition could potentially prevent successful colonization subsequent long distance dispersal, but is conceivably not limiting since it has been repeatedly shown that microspores are effectively attached to megaspores due to surface ornamentations (EL and CR pers. obs. and e.g., [24, 27, 28]). Details about isoetalean reproduction are, however, understudied. Self-compatibility may be common, as is e.g., suggested for the terrestrial polyploid *Isoetes durieui* [24]. Further, while both biotic and abiotic vectors have been proposed to aid in spore dispersal in *Isoetes*, it has been argued that known modes of dispersal are mostly confined to processes that can operate within the range of the population/species but not beyond it [24].

Thus, even if heterospory per se is not the confining problem regarding long distance dispersal, there are other potential limitations. Troìa [24] shows that only one species (*Isoetes durieui*) of at least three had successfully colonized volcanic islands located relatively close to the “main land” (Sicily), which indicates that long distance dispersal may be rare in *Isoetes.* Further, a four-year experimental study of *Isoetes lacustris* in central Europe [29] documented megagametophyte development in July through October, with sporeling development occurring during the subsequent spring(s). The authors find that the long lifecycle constitutes a limiting factor for sexual reproduction in the studied species, as does an observed requirement of relatively high temperatures during the germination process (≥ 12 °C for the megaspores). The latter conceivably constitutes another dispersal limitation, both geographically and concerning how deep fully submersed species are able to establish (although depth is also constrained by access to light). On the other hand, the distribution of some other species of *Isoetes* cannot be readily explained in any other way than effective long distance dispersal, e.g., the presence of *Isoetes* on comparatively young volcanic islands like Hawaii. The wide distribution range of some species may be another indication of successful long distance dispersal (although time and taxonomic choices are factors to consider as well).

### Phylogeny

Several previous studies have addressed the global phylogeny of *Isoetes*. Hickey [7] postulated, based on the presence of completely alate leaves that lack fibrous bundles, that three obligate aquatic South American species as well as members of the fossil genus *Isoetites* diverged first (as an unordered grade), leaving Indian species as sister to the remaining species of the genus [7]. The Indian species were characterized by having papyraceous “scales” (Hickey’s citation marks) while the remaining *Isoetes* species were united by presence of scales, phyllopodia and sporangial pigmentations [7]. However, even though morphology may be more informative for taxonomy and evolution in *Isoetes* than typically recognized [30], the simplicity of these plants clearly limits the possibilities of productively investigating relationships in *Isoetes* based on morphology alone, and subsequent phylogenetic studies have typically been based on molecular data [e.g., 23, 31–36. There are, in addition, several studies that have contributed important phylogenetic results at regional levels [e.g., 20, 28, 37–40].

While these previous efforts have meant substantial progress in our understanding of relationships within *Isoetes*, most of them are based on limited amounts of data. The same GenBank sequences have been repeatedly used in several studies, and information from the mitochondrial genome is lacking altogether. A few recent studies have utilized larger amounts of plastome and/or nuclear ribosomal data, but have typically used a more restricted species representation instead [35, 36, 39, 40]. Furthermore, an apparently complicated biogeographic pattern has emerged in several of these studies, which calls for further investigations. In an average angiosperm genus, such a lack of geographic phylogenetic structure would imply frequent recent and rapid dispersal beyond the geographic distribution of species, followed by colonization and speciation, i.e., dispersal being an important driver of (allopatric) speciation in the study group [41]. However, for a clade of lycopods like *Isoetes*, which potentially can be truly ancient, also other processes must be considered as potential explanations for an apparent lack of geographic phylogenetic structure. Using molecular dating, the (median) age of the crown group has been variously estimated to the latest Jurassic (c. 150 million years before present, Mya) based on chloroplast and nuclear ribosomal data [23, 34], to the mid-Paleogene (c. 45–60 Mya) based on nuclear data [35], and to the late Oligocene to early Miocene (c. 20–25 Mya) based on plastome data [35, 39]. Results in Wood et al. [35] indicate, however, that rate variation in the plastid genome hampers estimation of node ages.

The purpose of the present project is to further investigate evolutionary relationships in *Isoetes*, using newly produced molecular data and an extended sample of species compared to previous work, including species that have never before been included in a phylogenetic study. Because of the problems with morphological data (a simplistic morphological bauplan, with observed variation potentially having evolved in parallel) and the apparent lack of geographic phylogenetic structure in the genus as estimated in previous work, it is inadvisable to even provisionally infer the phylogenetic position of any species of *Isoetes* without results of phylogenetic analysis as a basis. We have chosen to only use newly produced data from the ingroup for the present study in order to evaluate data quality more rigorously than is possible when data from GenBank are being used. Our results reveal interesting news to science, among them the surprising new discovery of an “*Amborella* syndrome” in *Isoetes*, a single poorly known species being sister to the remaining clade.

## Results

In total, 702 sequences were newly produced for the present study. Number of analyzed samples, number of bases and number of variable characters (parsimony informative characters + singletons) in each region and in combined analyses, and model selection, are specified in Table 1.

**Table 1:** Data description and model specification

Markers	Samples*	Length (bp)	Variable characters (bp)*	Substitution model, maximum likelihood	Substitution model, Bayesian analyses
*ndhC-ndhK*	89(77)	1458	643(153)	TIM + I	GTR + I
*rbcL*	128(109)	1428	634(119)	TIMe + I + Γ	SYM + I + Γ
*rpoC1*	129(110)	3334	2249(391)	SYM + I + Γ	SYM + I + Γ
*trnV*^UCA^	104(99)	1313	399(245)	K3Pu + Γ	GTR + Γ
*ycf1*	129(110)	6538	4896(756)	TVMe I + Γ	SYM + I + Γ
*ycf66*	96(91)	1498	482(196)	HKY + Γ	HKY + Γ
Plastid markers^†^	132(113)	15,569	9303(1860)	TVMe I + Γ; GTR + I + Γ	SYM + I + Γ; GTR + I + Γ
Nuclear ITS	–(105)	783	–(372)	TPM3 + Γ	GTR + Γ
Total^‡^	130(111)	16,352	9675(2232)	TPM3 + Γ; GTR + I + Γ	GTR + Γ; GTR + I + Γ

*Values are given for the total dataset and (for *Isoetes* only).   ^†^Two partitions *(ycf1;* remaining plastid regions). ^‡^Two partitions (nuclear; plastid)

**Fig. 1:**
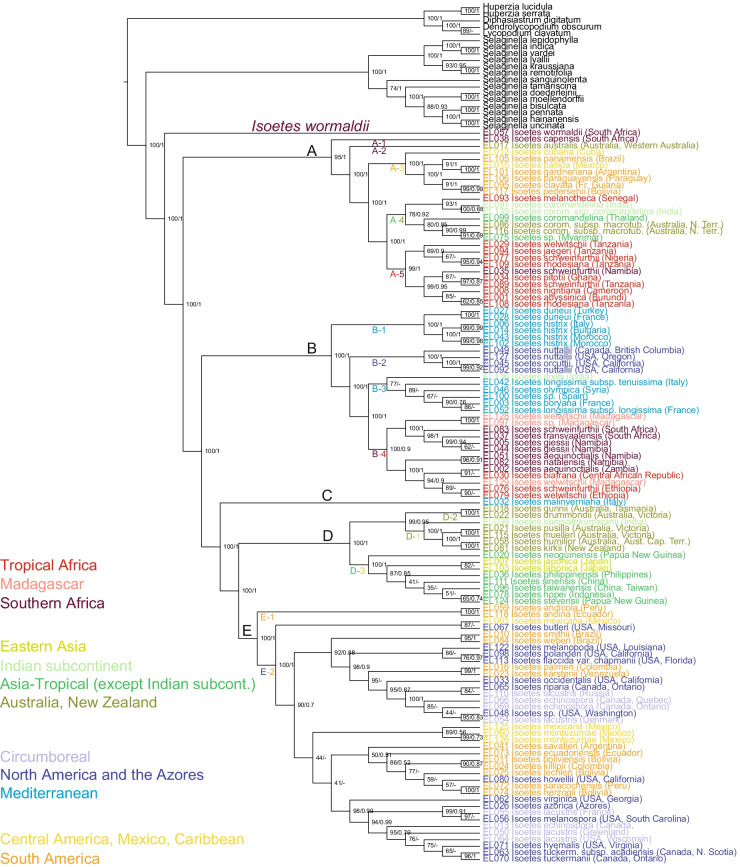
Phylogeny of *Isoetes* (Isoetaceae). Maximum likelihood analysis of plastid (*ndhC-ndhK, rbcL, rpoC1, ycf1, ycf66*, and *trnV*^UAC^ and its subsequent spacer) and nuclear ribosomal data (nrITS), but excluding nrITS data from outgroups and *Isoetes wormaldii* because of potential problems to infer positional homology (but see also Additional file 1: Fig. S3). Bootstrap support values and Bayesian posterior probabilities (as estimated in a separate analysis in MrBayes) are indicated on the tree as follows: maximum likelihood bootstrap / Bayesian posterior probability. Clade names A–E and their respective subclades are discussed in the text. Geographic distribution of species are indicated in color according to the legend to the left and following the World Geographical Scheme for Recording Plant Distributions [45]. An exception is the Mediterranean distribution, which refers to an occurrence in either one of the 22 sovereign countries in Europe, Africa and Temperate Asia that borders the Mediterranean Sea. Collection localities (country, and state for USA, Canada and Australia) of investigated samples are indicated in parenthesis to the right of the taxon names

Relationships among major groups of *Isoetes* (clades A–E; Fig. 1 and Additional file 1: Figs. S1–S3) are well-supported in all analyses (here defined as having a bootstrap support value ≥ 0.95 [42] and/or Bayesian posterior probability of ≥ 95 [43, 44]. Subclade division within these major clades [A: 1–5, B: 1–4, D: 1–3, E: 1–2, following Freund et al., reference 18] denotes clades that are well-supported in the combined analysis (Fig. 1) (and often in single genome analyses as well; Additional file 1: Figs. S1, S2), with the exception of clades A-1 and A-2 (for which only one sample each was included here), and clades A-4 and B-3 (which are poorly supported). Results among major clades are consistent between results obtained from plastid vs. nuclear ribosomal data, with the exception of the positions of two specimens, *Isoetes hypsophila* (sample EL123; Additional file 1: Fig. S4a) from China and one of the included representatives from southern Europe determined to “*I. velata”* (synonym; accepted name *I. longissima*) (sample EL120; Additional file 1: Fig. S4b). These specimens are successive sisters to clade D based on nuclear data (Additional file 1: Fig. S2) and to clade E based on plastid data (Additional file 1: Fig. S1), and they were not included in the combined analysis. Species determination of sample EL120 could, in addition, not be validated by us. Relationships within subclades of clades A-E may differ between results from plastid vs. nuclear data (Additional file 1: Figs. S1, S2; and descriptions below). Most of the apparently conflicting positions are, however, unsupported.

The combined analysis of plastid data and nuclear ribosomal ITS data (Fig. 1) is based on nrITS data from species of the *Isoetes* clades A-E. Nuclear ribosomal ITS data from *Isoetes wormaldii* and outgroups were excluded since their nrITS sequences were deemed too different from those of the remaining *Isoetes*. Alignment is, however, possible (although potentially with uncertainties regarding inference of positional homology) and a combined analysis based on plastid data and nrITS data was conducted, including nrITS data from *Isoetes wormaldii* and outgroups as well (Additional file 1: Fig. S3). Backbone results are well supported and consistent with those shown in Fig. 1 (where nrITS data for *I. wormaldii* and outgroups were excluded).

Geographic distribution of species (Fig. 1) are indicated following the World Geographical Scheme for Recording Plant Distributions [45]. An exception is the Mediterranean distribution, which refers to an occurrence in either one of the 22 sovereign countries in Europe, Africa and Temperate Asia that borders the Mediterranean Sea.

### Phylogenetic results—the combined analysis of plastid and nuclear ribosomal data (Fig. 1)

**Fig. 2:**
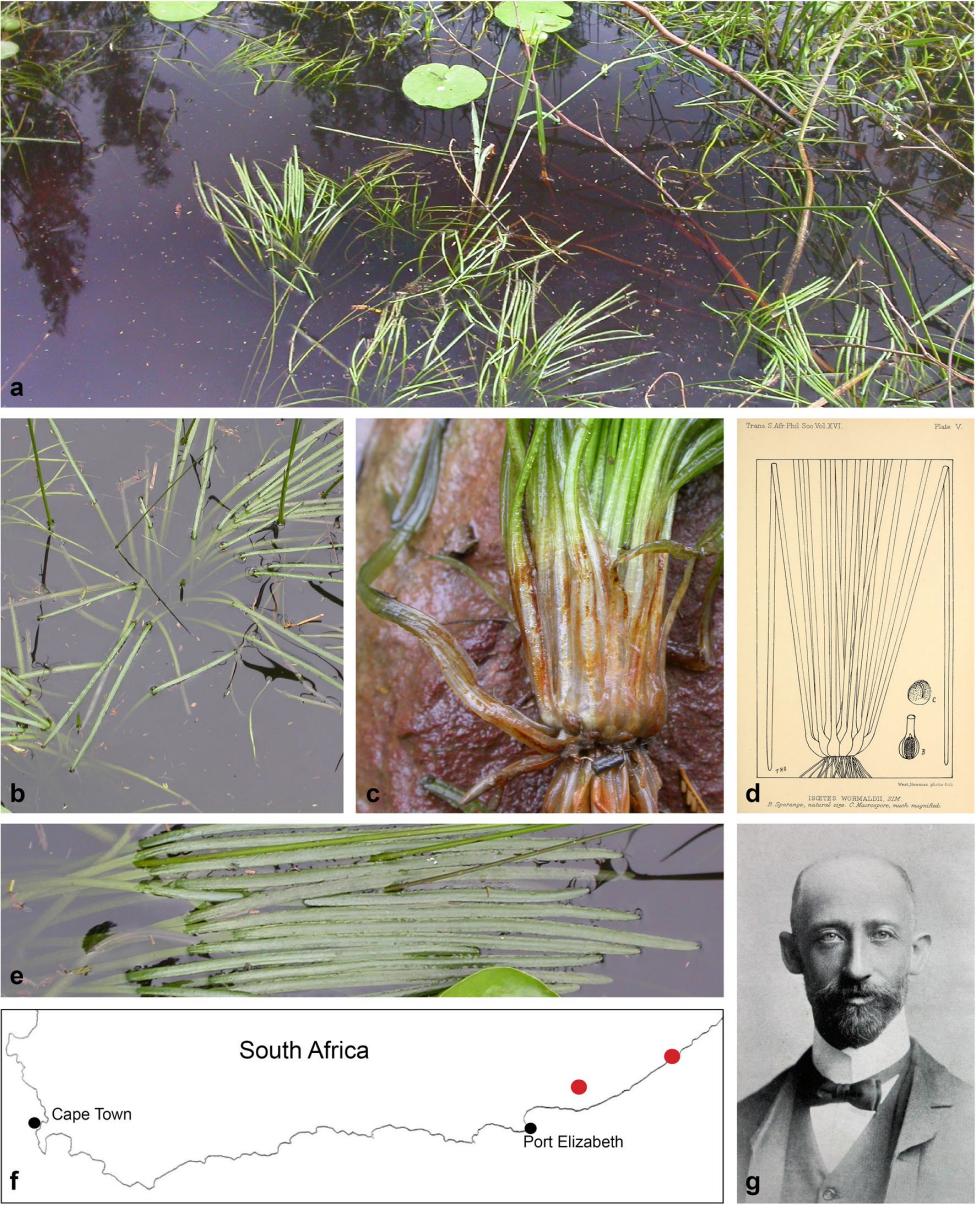
*Isoetes wormaldii* Sim. **a**–**c** *Isoetes wormaldii* in its natural habitat. Photos: Tony Dold (Rhodes University); **d** Drawing by Thomas Robertson Sim, reproduced with permission from the original publication by Sim 1905 [54]; **e** leaves with a single unbranched vein. Note the, for the genus, unusual leaf shape: flattened with a largely constant width to its rounded tip; **f** Map of southern South Africa. The two red dots indicate the only known populations of *Isoetes wormaldii,* which are located in the vicinity of the towns Makhanda (left) and East London (right) in the Eastern Cape, South Africa; **g** South African botanist Thomas Robertson Sim (1858–1938) (photographer unknown; source: Tr sim00.JPG—Wikimedia Commons)

The South African species *Isoetes wormaldii* (Fig. 2 and Additional file 1: Fig. S4c) is sister to the remaining genus (maximum likelihood bootstrap 100/Bayesian posterior probability 1) (Fig. 1). A clade that corresponds to clade A of Larsén and Rydin [23] is sister to the remaining genus. Within clade A (95/1), the South African *Isoetes capensis* (clade A-1) is sister to remaining species (100/1), followed by *Isoetes australis* (clade A-2). Remaining species in clade A (100/1) form two sister clades, of which clade A-3 comprises a set of South and Central American species (100/1). The other clade (100/1) comprises species from India, tropical Asia and Australia (clade A-4; 78/0.92), sister to a clade comprising species from tropical (to southern) Africa (clade A-5; 99/1). The tropical African species *I. melanotheca* is, however, nested in the A-4 clade, (which otherwise comprises species from India, tropical Asia and Australia), sister (93/1) to the Indian species *I. coromandelina* and *I. coromandelina* subsp. *coromandelina* (100/0.68). Support is generally high within the South-Central American clade (A-3), slightly lower in the Indian-tropical Asian-Australian clade (A-4) and the southern to tropical African clade (A-5).

A clade that corresponds to clade B of Larsén and Rydin [23] is sister to all species of the genus except *I. wormaldii* and clade A (100/1) (Fig. 1). Within clade B (100/1) the Mediterranean species *I. durieui* and *I. histrix* (clade B-1; 100/1) are sister to the remaining species (100/1). The North American west coast species *I. nuttallii* and *I. orcuttii* (clade B-2; 100/1) are sister to remaining species (100/1), which comprises two sister clades. One of them (clade B-3; 77/-) includes the Indian species *I. dixitii* sister to a group of Mediterranean species (89/-). The second (clade B-4; 100/0.9) comprises a set of species from southern (and tropical) Africa and Madagascar.

The Italian endemic *I. malinverniana* corresponds to clade C of Larsén and Rydin [23] and is sister to remaining species (100/1), which comprise two sister clades (100/1) corresponding to clades D and E of Larsén and Rydin [23].

Clade D (100/1) is divided into two subclades, one comprising species from Australia, New Zealand and India (clades D-1 + D-2; 99/0.95), and the other of species from eastern and tropical Asia (clade D-3; 100/1), within which one of the included species from New Guinea is sister to the remaining species (87/0.85).

Within clade E (100/1) two species from the northern Andes (clade E-1; *I. andicola* and *I. andina*, 100/1) are sister to remaining species (clade E-2; 100/1). Within clade E-2, which comprises species from South America, Central America and North America, two samples (one sample of *I. mexicana* and one of the south-central North American *I. butleri;* 87/-) constitute the sister of the remaining species (90/0.7). Support for relationships with clade E-2 is mostly low.

### Phylogenetic results—the combined analysis of plastid data (Additional file 1: Fig. S1)

As in the combined tree, the South African species *Isoetes wormaldii* is sister to the remaining genus (100/1) based on plastid data alone (Additional file 1: Fig. S1). Clade A sensu Larsén and Rydin [23] is sister to remaining species (100/1). Within clade A (97/0.96), the South African *I. capensis* (clade A-1) is sister to remaining species (92/0.94), followed by *Isoetes australis* (clade A-2). Remaining species in clade A (100/1) form two sister clades, of which one comprises a set of South and Central American species (clade A-3; 100/1). Its sister (97/1) comprises species from tropical (to southern) Africa, India, tropical Asia and Australia. Results in the South-Central American clade are well supported, whereas those of the latter clade are mostly poorly supported. Some results within this latter (tropical to southern African, Indian, tropical Asian and Australian) clade differ from those obtained in the analyses of nrITS (Additional file 1: Fig. S2) and of the combined analyses (Fig. 1 and Additional file 1: Fig. S3), for example the respective positions of *I. jaegeri* and *I. pitotii* and some samples of *I. schweinfurthii* and *I. welwitschii* but these differences are unsupported.

In clade B (100/1), the Mediterranean species *I. durieui* and *I. histrix* (clade B-1; 100/1) are sister to the remaining species (100/1). The North American west coast species *I. nuttallii* and *I. orcuttii* (clade B-2; 100/1) are sister to remaining species (clades B-3 + B-4; 100/1). Clade B-3 + B-4 comprises species from southern (to tropical) Africa, Madagascar, India and the Mediterranean region. Relationships within this clade may differ from those obtained from the combined analyses (Fig. 1 and Additional file 1: Fig. S3) and of nrITS (Additional file 1: Fig. S2), notably for example regarding the Indian species *I. dixitii*, but support values are generally low within this part of clade B.

The Italian endemic *I. malinverniana*, clade C sensu Larsén and Rydin [23], is sister to remaining species (100/1), which comprise clades D and E sensu Larsén and Rydin [23], as well as the Chinese *I. hypsophila* and one Mediterranean sample (“*I. velata*”; EL120).

Clade D (100/1) is divided into two subclades, one comprising species from Australia, New Zealand and India (clades D-1 + D-2; 95/0.82), and the other of species from eastern and tropical Asia (clade D-3; 100/1), within which one of the included species from New Guinea is sister to the remaining species (87/0.84).

The Chinese species *I. hypsophila* and one of the included representatives of the Mediterranean area (“*I. velata”*; sample EL120) are successive sisters to clade E. Support for *I. hypsophila* + *I. velata* + clade E is 100/0.89, for *I. velata* + clade E: 95/1, and for clade E: 95/1). Two species from the northern Andes, *I. andicola* and *I. andina*, (clade E-1; 100/1) are sister to remaining species of clade E (clade E-2; 100/1). Clade E-2, which comprises species from South America, Central America, North America, and the circumboreal region, contains two supported clades (both including species from South America, North America and the circumboreal region), and additional poorly resolved and supported diversity.

### Phylogenetic results—the analysis of nuclear ribosomal ITS (Additional file 1: Fig. S2)

The analysis of nuclear ribosomal ITS data, excluding nrITS data for *Isoetes wormaldii* and outgroups, was rooted on clade A, based on other results in the present study and previous work [23]. Within clade A (100/1), the South African *Isoetes capensis* (clade A-1) is sister to remaining species (99/1), followed by *Isoetes australis* (clad A-2). Remaining species in clade A (100/1) form two sister clades, of which one comprises a set of South and Central American species (clade A-3; 94/0.98). The other clade (clades A-4 + A-5; 99/1) comprises species from tropical (to southern) Africa, India, and Australia. Results within these clades may differ from those obtained based on plastid data (Additional file 1: Fig. S1) and the combined analyses (Fig. 1 and Additional file 1: Fig. S3), but are generally poorly supported.

In clade B (99/1), the Mediterranean species *I. durieui* and *I. histrix* (clade B-1; 100/1) are sister to the remaining species (99/1). The North American west coast species *I. nuttallii* and *I. orcuttii* (clade B-2; 100/1) are sister to remaining species. The remaining clade B (clades B-3 + B-4; 100/1) comprises species from southern (to tropical) Africa, Madagascar, India and the Mediterranean region. Relationships within this clade may differ from those obtained from the combined analyses (Fig. 1 and Additional file 1: Fig. S3) and of plastid data (Additional file 1: Fig. S1), notably for example regarding the Indian species *I. dixitii*, but support values are generally low within this part of clade B.

The Italian endemic *I. malinverniana* corresponds to clade C of Larsén and Rydin [23] and is sister to remaining species (94/1), which comprise clades D and E sensu Larsén and Rydin [23], as well as the Chinese *I. hypsophila* and one Mediterranean sample (“*I. velata*”; EL120).

Sample EL120 (“*I. velata”*, collected in the Mediterranean area) and the Chinese species *I. hypsophila* are successive sisters to clade D. Support for “*I. velata”* + *I. hypsophila* + clade D is 56/-, for *I. hypsophila* + clade D: 93/0.96, and for clade D: 96/1). Support for results within clade D is poor.

Clade E is well supported (98/1) but results within the clade are poorly supported.

Analyses of nuclear ribosomal ITS data including information from *I. wormaldii* and outgroups were conducted despite our conclusion that assessments of positional homology are uncertain. The resulting topology supported the sister relationships between the South African species *Isoetes wormaldii* and the remaining *Isoetes*. Other backbone results were also consistent with those presented in the present study, resolving clades A-E and their interrelationships, mostly with strong support. Consequently we performed a combined analysis of plastid and nuclear data, including nrITS data for *Isoetes wormaldii* and outgroups (Additional file 1: Fig. S3, details below).

### Phylogenetic results—the combined analysis of plastid and nuclear ribosomal data including nrITS data for *Isoetes wormaldii* and outgroups (Additional file 1: Fig. S3).

Results of the analysis of plastid and nuclear ribosomal ITS data, including nrITS data for *Isoetes wormaldii* and outgroups (Additional file 1: Fig. S3), were mostly congruent with results of the ditto analysis excluding nrITS data for *Isoetes wormaldii* and outgroups (Fig. 1). *Isoetes wormaldii* is sister to the remaining *Isoetes*, and clades A-E are with high support resolved as described above, as are the above-mentioned subclades of clades A-E. Clade A-4 is poorly supported, however.

## Discussion

To our great surprise, a poorly known and rarely discussed endemic species from South Africa is here shown to be sister to the entire remaining Isoetaceae (Fig. 1, Additional file 1: Figs. S1–S3). The result is strongly supported in all results, yet totally unexpected, and it can in many ways be argued to be the lycopod equivalent of the 1999 discovery that the poorly known New Caledonian endemic *Amborella tricopoda* was sister to the remaining angiosperms [46, 47, 48, 49]. The conclusion was a consequence of previous tentative indications [50, 51] that called for further investigations of *Amborella’s* systematic position. In our case, no previous indications exist; *Isoetes wormaldii* has rarely been investigated for any purpose and has never before been included in a phylogenetic study. It was included in the present study because we aimed for a taxon sampling as broad as possible, and material was available to us. The result is thus yet another reminder that it is very difficult to predict the approximate systematic position of an *Isoetes* species that has not been included in a phylogenetic analysis. Surprising phylogenetic results that contradict intuitive assumptions (e.g., based on geographic proximity of species) have repeatedly been uncovered in studies of *Isoetes* based on molecular data*,* beginning with Hoot & Taylor [31] and Rydin & Wikström [32] who showed that North American species are not monophyletic and that some South American species are closer related to some African species than to other South American species.

### *Isoetes wormaldii*

*Isoetes wormaldii* (Fig. 2a–e; Additional file 1: Fig. S4c) appears to possess some potentially interesting morphological oddities that are worth mentioning. It is an extremely rare, decreasing, and critically endangered species, known only from a few localities in the Eastern Cape region of South Africa [52, 53]. It was first discovered submersed in ponds in the area of East London in southeastern South Africa [54–56], and it is in addition reported from a few localities in the area of the town Makhanda (formerly known as Grahamstown) (Fig. 2f), where it grows submersed in fresh water ponds and slow-flowing streams [53] (Fig. 2a, b). Each subpopulation is very small, comprising only around a dozen plants, and populations are reported to disappear when depraved of grazing by cattle [53]. Consequently, it is sensitive to exploitation of habitats and has strongly declined due to agricultural cultivation expansion and urbanization [53]. However, monitoring indicates that spores may germinate after years of dormancy, since new plants suddenly can reappear after being reported missing [53].

**Fig. 3:**
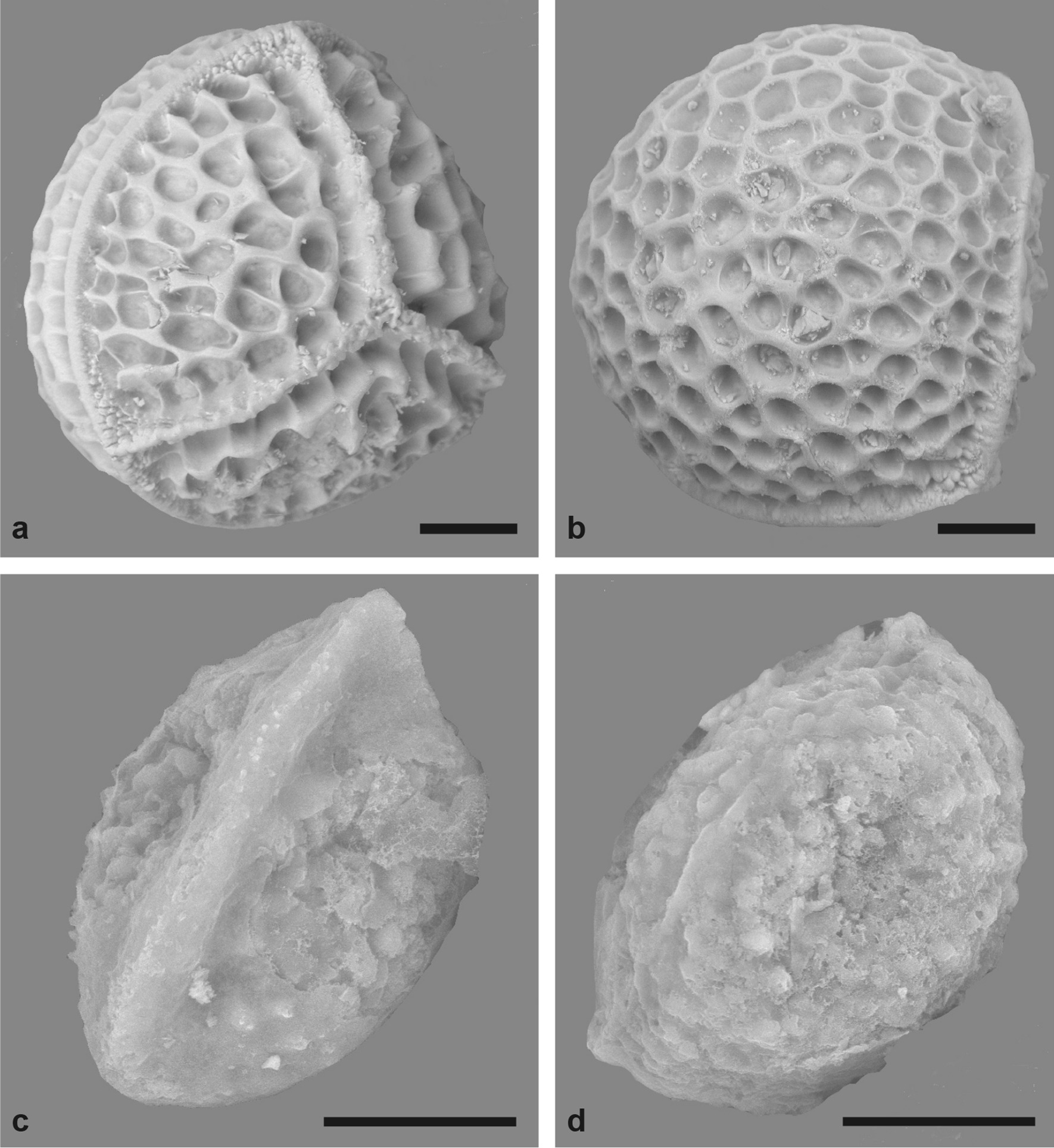
Spores of *Isoetes wormaldii* (Pocock 20009 [BM]). **a** Megaspore, proximal view (slightly to the side); **b** Megaspore, distal view (slightly to the side); **c** Microspore, proximal view (slightly to the side); **d** Microspore, distal view (slightly to the side). Scale bars: **a**, **b** 100 µm; **c**, **d** 10 µm

The species was formally described by Thomas Robertson Sim (Fig. 2g) in 1905 [54], and named after W. H. Wormald who first discovered the plant in 1893 in ponds around East London, South Africa [54, 55]. According to the original description [54], its leaves are relatively long, rising to the surface and then floating (see also Fig. 2a, b). When they occur in deep water, the leaves may grow up to a length of 45 cm [55]. The leaves were said to have three veins, one central and two marginal [54], but this was clearly a misinterpretation (since lycopod leaves are characterized by having a single unbranched vein) and subsequent work showed that leaves of *I. wormaldii* have no more than a single central vascular strand [4, 55]. The leaves of *I. wormaldii* are “somewhat flattened” (in transverse section; Fig. 2c–e) and “hardly narrowed to the rounded point” [54] (Fig. 2e), features we find unusual in *Isoetes*. *Isoetes* leaves are generally described as subulate [e.g., 5, 6], awl-shaped with reduced lamina (ala), but Hickey [7] argued that a few South American species (*I. bacculata*, *I. bradei*, *I. gigantea*) and fossils of *Isoetites* have laminate leaves [7 and references therein]. The same is thus true for the South African *I. wormaldii*. In *I. wormaldii,* the flattened appearance is at least in part caused by size reduction of the air channels (lacunae). Two of the four longitudinal air channels that are normally present in leaves of *Isoetes* (the two adaxial channels) are poorly developed in the basal parts of the leaves and distally gradually even more so, to completely disappear at the tip of the leaves [55], giving the leaves their flattened appearance as seen in transverse section. Reduction of the number of air channels are otherwise rarely reported for leaves of *Isoetes* [but see Troia and Raimondo, reference 9], as is laminate leaves with a flattened shape as seen in transverse section. Such leaves are according to Hickey [7] possibly unknown in *Isoetes* with the exception of *I. bacculata*, *I. bradei*, *I. gigantea*, and are not described for the other South African species discussed by Duthie [55]. Flattened, apparently laminate leaves occur, however, in isoetalean fossils such as the Early Triassic *Isoetes beestonii* [Fig. 3:6 in reference 57] and the Middle Jurassic *Isoetites rolandii* [Figs. 1 and 6 in reference 58]. Hickey [7] argued that *Isoetites* and the three extant South American species *I. bacculata*, *I. bradei* and *I. gigantea* represent unrelated but ancestral lineages, possibly (successive?) sister lineage(s) to the remaining extant clade. While evolution of leaf shape in *Isoetes* is complex, it is interesting to note that the laminate/alate *I. wormaldii* is sister to the remaining species of the genus (Fig. 1). Previous work [23, 34] has shown that the laminate/alate *I. bradei* and *I. gigantea* are nested within clade A [sensu Larsén and Rydin, reference 23], which is sister to the remaining genus except *I. wormaldii*, and referred to as a possible “Gondwana clade” [33 and subsequent work].

The corm-like stem of *Isoetes* becomes lobed at the base [4, 55, 59, 60]. While the number of lobes were variable in extinct isoetaleans, modern day *Isoetes* are 2-lobed or 3-lobed (sometimes with ontogenetic modifications) [3–5, 60, 61]. Based on ancestral state reconstruction on a sample of phylogenetic trees, Freund et al. [18] found that the 3-lobed condition is ancestral. Our results, placing the 3-lobed *I. wormaldii* [4, 54, 55] as sister to the remaining genus, support the conclusions in Freund et al. [18].

The megaspores of *I. wormaldii* are of the typical *Isoetes* type [i.e., trilete with a distinct equatorial ridge, 4], but the ultrastructural ornamentation of the megaspores is reticulate (Fig. 3a, b) [4, 55] in a distinct pattern we have not seen otherwise documented neither in the literature nor in our own studies. The microspores are monolete and the proximal ridge is prominent (Fig. 3c, d). There are in addition two less prominent distal ridges and the microspores were said to be “3-ridged” in the original description [54]. This should not be misunderstood as a trilete condition; all extant species of *Isoetes* have monolete microspores [27], and this is thus also true for *I. wormaldii* (Fig. 3c, d). Trilete microspores occur in the living sister group *Selaginella* [62], and in some extinct members of the isoetalean lineage. Isoetaleans with trilete microspores are documented through time, e.g., in the Late Devonian *Clevelandodendron ohioensis* [63], in the Triassic *Isoetes beestonii* [57] and *Pleuromeia rossica* [64], but during the Mesozoic, trilete microspores become more rare in the Isoetales and an evolutionary trend from trilete to monolete microspores has been hypothesized for the isoetalean lycopsids through the Mesozoic [10, 16, 60]. It should be noted, however, that interpretations of spore evolution in the Isoetales are complicated by the fact that the outermost layer of the spore, a silicified perispore, may not survive fossilization [60, 65, 66].

### The genus-wide phylogeny

Remaining phylogenetic results (Fig. 1) are in agreement with previous work with comparatively extensive global sampling of the genus [e.g., 23, 31–34]. However [and in line with the relatively few previous studies that have included more than one sample per species, e.g., 34], our results clearly indicate the need for extensive alpha-taxonomic work on *Isoetes.* Some of the most widespread species (i.e., the circumboreal *I. lacustris* and *I. echinospora*, and the African *I. schweinfurthii* and *I. welwitschii*) are clearly not monophyletic. These species have a complicated taxonomic history, often with ample described taxa that are currently considered synonymous to these species (see examples from the present study in Additional file 2: Appendix S1). To understand the underlying biology requires extensive investigations, including morphological studies of a large set of specimens. It appears likely that such future work will lead to revisions of current species delimitations, at least for these presumably widespread species.

*Clade A*. Clade A (Fig. 1) contains species from the southern hemisphere*. Isoetes capensis* from South Africa is here sister to the remaining species of clade A, and previous work has shown that *I. capensis* groups with additional South African species, i.e., *I. stellenbossiensis, I. stephanseniae, I. toximontana* [23, 33–35] and *I. eludens* [34]. Several of them are seriously threatened. *Isoetes capensis* of the Western Cape is considered endangered and declining [67], and *I. stephanseniae* is, like *I. wormaldii*, critically endangered [68].

*Isoetes australis* from western Australia is clearly not closely related to other Australian species, and differs from them in several respects. It further possesses some features that are unique or rare within the entire genus, e.g., regarding its anatomy and distichous leaf arrangement [69]. It differs from most (but not all) other Australian species, and from most (but not all) other species in clade A, in that its corm is 2-lobed, not 3-lobed [18, 69]. Williams [69] argues in the original description of the species that its small size, distichous phyllotaxy, and unique anatomy indicate a permanently juvenile condition compared to other species of *Isoetes*.

A diverse and broadly distributed clade of South American and Central American species occurring from Cuba and Mexico in the north to Argentina in the south is also included in clade A (A-3). Knowledge of diversity, phylogeny and biogeography of South American species of *Isoetes* has increased dramatically as a consequence of recent work by Pereira and colleagues [34, 36, 39, 40], including description of new species [e.g., 70, 71, 72] as well as studies at the population level [e.g., 73, 74]. The South-Central American clade of clade A (A-3) comprises at least 17 species [the present study and results in previous work, references 23, 34, 36, 39].

The South-Central American clade is sister to a clade of Indian/tropical Asian/Australian species (clade A-4) plus a mostly tropical African clade (clade A-5). The former clade (A-4) includes both subspecies of *I. coromandelina* (i.e., the Indian/subcontinental *I. coromandelina* subsp. *coromandelina* and the northern Australian *I. coromandelina* subsp. *macrotuberculata*). It is surprising to note that a West tropical African species is nested within this otherwise Asian/Australian clade: *I. melanotheca*. This species has to our knowledge not been included in previous phylogenetic work and its position needs to be confirmed using additional representatives of the species than the single sample used here. Sister to the Indian/tropical Asian/Australian/tropical African clade is a clade (clade A-5) that comprises a number of species from southern (and tropical) Africa and Madagascar (i.e., *I. welwitschii, I. schweinfurthii, I. jaegeri, I. nigritiana, I. pitotii, I. abyssinica, I. rhodesiana*), some of which are relatively widespread as currently circumscribed. Our results (the position of sample EL035) as well as previous work [32, 34] tentatively indicate that *I. kersii* is included as well. However, phylogeny and species delimitations of African species of *Isoetes* need more research and probably some alpha-taxonomic revision. Results within clade A-5 are partly poorly supported and may conflict between results from plastid and nuclear data (although conflicting results are unsupported). *Isoetes rhodesiana* and *I. kersii* are considered synonymous with *I. schweinfurthii* [3], but our results show that *I. schweinfurthii* is non-monophyletic, and the same holds for *I. welwitschii*. Further studies and taxonomic revision should be based on a substantially expanded sample of African *Isoetes*.

*Clade B.* The presence of clade B (Fig. 1) was indicated already in early work based on molecular data [31], but it has nevertheless remained poorly known until recently. Several studies did not include any representatives of the clade [32, 33]. Based on results of the present study and previous work with extensive global sampling of the genus [23, 34], it is evident that clade B has a nearly worldwide distribution with representatives from the Mediterranean region (clades B-1 and B-3), North America (clade B-2), India (clade B-3), and southern (to tropical) Africa and Madagascar (clade B-4). The European species of clade B are thus resolved in two groups (clades B-1 and B-3) that correspond respectively to the *Isoetes histrix* group and the *Isoetes longissima* group of Troía et al. [20]. We show that *I. boryana* and *I. longissima* subsp. *tenuissima* are included in the *Isoetes longissima* group, as predicted by Troía [e.g., 75]. It appears, however, uncertain if *I. longissima* represents a single species [Fig. 1 of the present study as well as Fig. 2 in reference 20].

The biogeographical history of clade B is not readily understood. Based on the phylogenetic pattern Larsén and Rydin [23] speculated that clade B is the Laurasian equivalent to the (possibly) Gondwanan clade A, and results in Pereira et al. [34] resolved the clade (B) as having a European/North African ancestry. However, the inclusion of a substantial number of African species, and an Indian species in clade B (Fig. 1) would, if vicariance is assumed the main biogeographic process responsible for the observed pattern, rather point to a Pangean origin of the clade, something that is refuted by the hereto estimated crown age for clade B of the earliest Paleogene [23, 34], or younger [35, 39]. While more recent dispersal processes are evident in the clade, e.g., between southern Africa and Madagascar, and between Europe and northern Africa (Fig. 1), the large-scale phylogenetic pattern in clade B may potentially indicate an older clade with substantial extinction [for example of elements of the early Cenozoic Tethys flora as discussed in 23], despite an apparent incongruence with previously estimated node ages.

*Clade C.* As in previous work [23, 32–36, 39], the Italian endemic *I. malinverniana*, with a critical conservation status [76], is sister to a large clade comprising species from Asia, Australia and New Zealand (clade D) as well as a clade that includes American species and species with a circumboreal distribution (clade E) (Fig. 1). Larsén and Rydin [23] included several samples of *I. malinverniana* and the results indicated monophyly of the species. Bolin et al. [28] showed that the southwest Asian *I. anatolica* is sister to *I. malinverniana*. The morphological similarities mentioned for the two [3-lobed corm and a lack of velum, 28] are, however, not unique to these two species but occur for example also in several South African species including *I. wormaldii* [55].

*Clade D.* Clade D comprises a mostly Australian clade (clade D-1), that also includes the Indian *I. sampathkumaranii* and species from New Zealand [i.e., *I. kirkii* and in addition *I. alpina* as shown in previous work, reference 23]. While *I. australis* of clade A is restricted to western Australia and *I. coromandelina* subsp. *macrotuberculata* (also of clade A) to northern Australia, the Australian species of clade D are more broadly distributed, or restricted to the southeastern parts of the continent. Clade D-1 is sister to an Australian clade (D-2) with apparent southern to Tasmanian distribution. The species of clade D-3 are widely distributed in eastern and tropical Asia (Fig. 1) [see also reference 23 for extended sampling]. Studies have indicated that the Chinese species *I. hypsophila* is sister to the remaining clade D [e.g., 33] and it has been used as an a priori decided outgroup in studies of Asian *Isoetes* [e.g., 77, 78]. Here, we removed *I. hypsophila* from the final combined analyses because our single sample of the species (Additional file 1: Fig. S4a) displays distinctly different phylogenetic positions as analyzed using plastid data and nuclear ribosomal data (Additional file 1: Figs. S1, S2). While the “sister to clade D hypothesis” is supported in the analysis of nuclear ribosomal ITS (Additional file 1: Fig. S2), the species is sister to clade E based on plastid data (Additional file 1: Fig. S1). Tentative comparison indicates that our sequences of *I. hypsophila* are congruent with data from this species that has been previously deposited on GenBank. It is nevertheless difficult to speculate regarding the reason for this apparent cytonuclear discordance; that the species may be of hybrid origin is only one of several possible biological and methodological explanations.

*Clade E.* The two Andean species *I. andina* and *I. andicola* (clade E-1) are here strongly supported as sister to the remaining clade E (clade E-2) (Fig. 1). *Isoetes andicola* was sister to the remaining species in the equivalent of our clade E in a recent study [36] based on large amounts of data (plastome data) although with a more restricted sample of taxa than included here. The result is interesting because *I. andicola* was formerly placed in its own genus (based on stem morphology) [79]; however, our results show that the species is not the lone sister species of the remaining clade E but part of a clade that comprises at least one additional species (*I. andina*; Fig. 1).

The remaining clade E (clade E-2) comprises a substantial diversity of American species, and in addition some species with circumboreal distribution extending through Canada, Greenland, Scandinavia and Russia. One of these species is *I. lacustris*, and the here included samples of it from Russia, North and central Europe, Greenland and the United States do not form a clade. The same holds for our included samples of *I. echinospora*, which is present in two supported clades within clade E. Transitions between North America, Central America and South America appear to have occurred repeatedly in clade E but the poor resolution in the clade prevent further conclusions. The lack of resolution in this clade may at least partly be a result of a high prevalence of polyploids/hybrids and subsequent reticulate evolution, which appear common in *Isoetes*, at least in American species (i.e., our clade E) [22, 40, 80] but may also be due simply to lack of sufficient variation in the molecular markers used here. Using the entire plastome for phylogenetic inference in clade E has for example proven successful [36, 38], although allopolyploidy/hybridization likely will go undetected unless complemented with other sources of data.

As with *I. hypsophila* discussed above, a sample of “*I. velata”* collected in Portugal (EL120; Additional file 1: Fig. S4b) was removed from the combined analyses because of conflicting positions in results based on plastid vs. nuclear ribosomal data (Additional file 1: Figs. S1, S2). It is either well-supported as sister to clade E (plastid data, Additional file 1: Fig. S1), or the unsupported sister to clade D and *I. hypsophila* (nuclear ribosomal ITS, Additional file 1: Fig. S2). It further seems clear that the sample is misidentified; other samples of *I. longissima* (the accepted name of the synonym *I. velata*) are resolved in clade B. Our investigations do not provide a straightforward indication on what species this sample may instead represent. Most European species belong in two separate clades within clade B and are discussed above, but a few species belong in clade E, (apart from the widespread *I. lacustris* and *I. echinospora* also *I. azorica*, Fig. 1). Previous results [20] show that additional European species belong in clade E (i.e., *I. delilei, I. phrygia* and *I. todaroana*), but their relationships to the vast diversity of species in clade E are so far understudied.

In some previous work [23, 33, 34, 37], sequences produced from one-two samples of *I. histrix* have been used and these samples were resolved in the equivalent of clade E (whereas other samples of the species are resolved in the equivalent of clade B, see also above). This apparent contradiction regarding the phylogenetic position of *I. histrix* was, however, recently resolved by Troía et al. [20] who identified that this material sequenced by Hoot and colleagues actually represents another species, *I. phrygia*. The material was originally collected in Greece by a colleague of ours, Dr. Hans-Erik Wanntorp (Wanntorp NR5350). We have previously used an *rbcL* sequence produced from other material sampled in Greece by Dr. Wanntorp in two of our studies [23, 32], samples that also fall in clade E in those studies. Although not produced from the same plant material as used by Hoot and colleagues, it appears reasonable to believe that all these plants come from the same locality, and we therefore conclude that the *rbcL* sequence of “*I. histrix*” originally produced by one of us (CR) during work with Rydin and Wikström [32] actually represents the species *I. phrygia*, not *I. histrix*.

### Biogeography and node ages

Biogeography, node ages, and influential processes such as spore dispersal ability and speciation have repeatedly been discussed for *Isoetes*, based on morphology and/or chromosome data [e.g., 4, 8, 21, 24–26, 28], molecular data [e.g., 20, 32, 33, 78], dated molecular phylogenies [e.g., 23, 35] and dated phylogenies in combination with biogeographic analyses [e.g., 34, 37, 39]. Topological results of phylogenetic analyses of *Isoetes* are not easily translated into self-evident biogeographic patterns and processes. Species occurring in southern to tropical Africa fall into five major clades, Indian species are resolved in three major clades, Australian and tropical Asian species are present in three and two clades, respectively, and South American species occur in at least three clades. The same is true for the Northern Hemisphere; both European and North American species are each placed in at least three major clades. Furthermore, because the crown group *Isoetes* belongs to the ancient isoetalean lineage, it has the potential to be truly ancient. Vicariance can therefore not be a priori excluded as a potential explanation for the observed topological patterns. However, evolution is a continuous process and even if ancient major vicariant events have affected the phylogeny of *Isoetes*, more recent dispersal has too. A study of Mediterranean species indicated that long-distance dispersal followed by successful colonization may be uncommon in the genus [24]. On the other hand, with time even rare events may accumulate and become part of the evolutionary history of a group. The distribution patterns of some species indicate that long distance dispersal may have successfully occurred in *Isoetes*, and a strong dispersal capacity appears evident in at least some species (e.g., *I. azorica*, *I. hawaiiensis, I. japonica* and species of tropical Asia*,* and potentially also in the circumboreal *I. lacustris* and *I. echinospora*).

Studies on the biogeographic history of a group of organisms should be set up as testable hypotheses [81]. The potential effect of vicariance can for example be explicitly tested for, using analyses of divergence times of clades where the result may reject or not reject the hypothesis that a certain vicariance event caused (explains) a certain clade divergence [81]. Therefore, rigorous results on nodes ages appear as the most important first step for a better understanding of historical biogeography in *Isoetes*. Unfortunately, robust results on nodes ages have proven difficult to accomplish for *Isoetes*. Larsén and Rydin [23] and Pereira et al. [34] found similar node ages with, for example, a median age of the crown group *Isoetes* of around 150 Mya, i.e., before the final rifting of the Gondwana. By contrast, results in Wood et al. indicate that extant diversity originated approximately 100 million years later [35] or even some 130 million years later based on plastome data [35, 39], thus at a time when the continents were approaching their current positions. All these results are in turn in opposition with those of Kim and Choi [37], who report much older node ages (e.g., clade BCDE originating at the Triassic-Jurassic border, c. 250 Mya).

Analytical methodologies and data utilized were not identical in these studies, which may explain deviating results, and we agree with Wood et al. [35] who conclude that both the use of appropriate fossil calibrations and the choice of molecular data are very important and will have critical impact on estimated dates [35]. It is nevertheless clear that it is difficult to accomplish a robust age estimate of the crown group of *Isoetes* (NW, EL, CR, work in progress), which complicates the use of node ages to infer biogeographic processes responsible for the observed phylogenetic and distributional patterns in *Isoetes*. While for example the topological results in the *Isoetes* clade A (Fig. 1) indeed bring to mind an ancestry of ancient Gondwana distribution(s), including several subclades with intriguing patterns (e.g., a southern African species sister to two Indian species), it is not possible to exclude other processes at this point. Long-distance dispersal followed by colonization and allopatric speciation must also be considered, for example by testing the hypothesis of a causal effect resulting from the atmospheric/oceanic West Wind Drift, a Cenozoic process that will result in (unidirectional) dispersal patterns and topologies that are different from those expected as a consequence of allopatric speciation following the sequential break-up of the Gondwana continent [e.g., 82, 83] during the Mesozoic. Furthermore, and as is speculated on above, apparent biogeographical patterns can be misleading due to substantial extinction [81], not least in a potentially old genus like *Isoetes*. Recent methodological advances are promising regarding possibilities to take extinction more explicitly into consideration in biogeographic studies [84], but to successfully utilize such methodological progress in studies of *Isoetes* will require that fossils can be unambiguously placed, phylogenetically, within the extant clade in the future.

## Conclusions

Our work provides new information on global diversity in Merlin’s grass and show, in line with previous work, that their biogeographical history appears complex, possibly including a mixture of ancient (Mesozoic to early Cenozoic) and more recent (Quaternary) processes. An utterly unexpected outcome of our work is the here detected sister-relationship between the poorly known and rarely discussed South African species *Isoetes wormaldii* and the remaining genus (Fig. 1 and Additional file 1: Figs. S1–S3). Moreover, our studies of *I. wormaldii* reveal that this species is strikingly different from other species of *Isoetes*, both regarding sequence divergence (see e.g., the phylograms in Additional file 1: Figs. S1–S3) and morphology. As discussed many times [e.g., 23, 32, 39, 85], sequence divergence in the remaining *Isoetes* is very low even in markers that are otherwise typically useful for analyzing species level relationships, such as nrITS, and this has hampered phylogenetic reconstruction. Here we discovered that the nrITS sequence of *Isoetes wormaldii* is so different from that of other species in the genus that we felt compelled to remove it from our analyses (but see Additional file 1: Fig. S3). The implications these finding will have on futures studies on node ages of and within the crown group have not gone unnoticed by us.

Also from a morphological point of view does this rare species stand out as different from most species in the genus, with laminate (non-subulate) leaves that float on the water surface (Fig. 2), reduced air channels, and a megaspore (perhaps also microspore?) ultrastructure that appears rare to us (Fig. 3).

We have not conducted explicit biogeographic analyses but southern Africa/South Africa appears to be a candidate as the ancestral area of the extant clade, and species of this region may contain (additional) unique morphological and/or genetic diversity. A more complete review of conservation status of *Isoetes* is beyond the scope of our work, but we notice that many of the South African species are extremely rare, decreasing and threatened. *Isoetes wormaldii* (Eastern Cape) is critically endangered, as is the Western Cape endemic *I. stephanseniae* of clade A-1 [23]. *Isoetes wormaldii* and *I. stephanseniae* are apparently at the brink of extinction, with only a few small populations having ever been known, one (each) of which may already be lost due to agricultural development and urbanization [53, 68]. The situation is only marginally better for *Isoetes capensis* (clade A-1), with a limited distribution in the Western Cape; it is considered endangered and declining [67]. Since these species belong to species-poor clades, that are sister to the remaining genus, and the remaining clade A, respectively, they are lone representatives of clades that conceivably were more diverse in the past. To save these species for the future, i.e., preserve the habitats in which they still occur, appears to us to be of highest possible priority.

## Methods

### Taxon sample and data production

We utilized herbarium material for the present study and we aimed at including as many species as possible, limited only by material availability and quality of herbarium material from this group with worldwide distribution. We deemed it particularly important to sample African specimens as less work has previously been done on species of *Isoetes* with that geographic distribution. Whenever possible, we included more than one sample from each species. In total, 113 samples of *Isoetes* were utilized representing 81 taxa and four samples undetermined to species. Species from the remaining Lycopsida were used as outgroups. Taxon names, voucher information, geographic distribution of the species and collection locality of the vouchers, are given in Additional file 2: Appendix S1 together with GenBank accession numbers.

We selected six plastid markers (*ndhC-ndhK, rbcL, rpoC1, ycf1, ycf66*, and *trnV*^UAC^ including its subsequent spacer), and the nuclear ribosomal internal transcribed spacer (nrITS) for the present study. All gene regions were alignable across the entire data set except nrITS, for which assessments of positional homology was difficult to infer when including *Isoetes wormaldii* and outgroups in the alignment. Nuclear ribosomal ITS was used only for the ingroup (except *Isoetes wormaldii*) in final analyses, but analyses including nrITS also for *Isoetes wormaldii* and outgroups were conducted at earlier stages of our work (results of the combined analysis of plastid and nuclear data, including nrITS data from *I. wormaldii* and outgroups are presented in Additional file 1: Fig. S3). Of the six regions we selected from the plastid genome, only one (*rbcL*) has been widely used for inferring the phylogeny of *Isoetes* in previous work, and few studies have included a comprehensive sample of taxa from the entire genus. Primers for the plastid regions except *rbcL* were newly produced for the present study based on entire chloroplast genomes produced for a set of species of *Isoetes* as part of other ongoing work (Wikström et al. in progress). All primers are specified in Additional file 2: Appendix S2. Outgroup sequences were downloaded from GenBank [86] and treated at the species level rather than specimen level (as for the ingroup). The *trnV*^UAC^ and *ycf66* regions are missing for *Selaginella.*

Extraction of total genomic DNA was performed according to the cetyltrimethylammonium bromide (CTAB) method [87, 88], and purified using a QIAquick PCR Purification Kit (Qiagen, Sweden). PCR reactions were conducted using standard procedures outlined elsewhere [e.g., 89], and optimized for the here utilized primers and extractions. Sequencing was performed by the Macrogen Sequencing Service (Amsterdam). Obtained reads were assembled in Geneious version 9.1.8 [90]. Alignment was conducted using software MAFFT v. 7 [91] with the algorithm G-INS-i with a variable scoring matrix, and subsequent corrections by eye.

### Phylogenetic analyses

ModelFinder [92] as implemented in the IQ-TREE web server [93, 94] was used to find the best fitting models and partitions [92, 95]. Best fitting substitution models (the criteria AIC, AICc and BIC gave similar scores) for individual gene regions and combined datasets are given in Table 1. Supported topological conflicts were not detected and a combined analysis of all the plastid data (two partitions: *ycf1*; remaining plastid regions) was therefore conducted. Subsequently, an analysis combining plastid data with nrITS was conducted. For the combined plastid and nuclear analysis, two partitions were applied (one for nrITS and one for the plastid regions). Maximum likelihood analyses were conducted on the IQ-TREE web server [93, 94]. Bootstrap support values were obtained using Ultrafast bootstrap [UFBoot, 42, 96] as implemented in IQ-TREE [97] with number of bootstrap alignments set to 1000, maximum likelihood iterations set to 1000, minimum correlation coefficient set to 0.99 [96] and other settings at default values [92]. Bayesian inference of phylogeny was conducted in MrBayes v. 3.2.7a [98, 99] at the Cipres Science Gateway [100] with default prior probabilities and employing the respective models implemented in MrBayes that are most similar to those estimated as best fit for each data set using ModelFinder [92] (Table 1). Two parallel runs of four chains each were run for 20 million generations, with a sample frequency of trees and parameter estimates of 1000. Convergences of runs and suitable burn-in were assessed in Tracer v. 1.7.2 [101], and using the PSRF convergence diagnostic in MrBayes. Bayesian posterior probability values were calculated after discarding the first 10% of the trees and parameters as burnin.

## Supplementary Information

**Additional file 1: Fig. S1. **Maximum likelihood analysis of plastid data (*ndhC-ndhK, rbcL, rpoC1, ycf1, ycf66*, and *trnV*UAC and its subsequent spacer). Bootstrap support values and Bayesian posterior probabilities (as estimated in a separate analysis in MrBayes) are indicated on the tree as follows: maximum likelihood bootstrap (BS) / Bayesian posterior probability (PP). Clade names in green (A-E), subclade names in color following the scheme in Fig. 1 of the main text, and sample names in red are discussed in the text. Phylogram to the left with branch lengths upscaled 10 times and outgroups removed. **Fig. S2. **Maximum likelihood analysis of nuclear ribosomal data (nrITS). Bootstrap support values and Bayesian posterior probabilities (as estimated in a separate analysis in MrBayes) are indicated on the tree as follows: maximum likelihood bootstrap (BS) / Bayesian posterior probability (PP). Clade names in blue (A-E), subclade names in color following the scheme in Fig. 1 of the main text, and sample names in red are discussed in the text. Phylogram to the left. **Fig. S3. **Maximum likelihood analysis of plastid (*ndhC-ndhK, rbcL, rpoC1, ycf1, ycf66*, and *trnV*UAC and its subsequent spacer) and nuclear ribosomal data (nrITS). This analysis is equivalent to that depicted in Fig. 1 of the main text, with one exception: here, nuclear ribosomal data is also included for *Isoetes wormaldii *and eight outgroup taxa representing the Selaginellaceae and Lycopodiaceae. Despite potential difficulties to infer positional homology when aligning the nrITS sequences of outgroups and *Isoetes wormaldii *with those of the remaining *Isoetes*, most results are identical to those shown in Fig. 1. Bootstrap support values are indicated on the tree. Clade names in purple (A-E) and subclade names in color following the scheme in Fig. 1 of the main text, are discussed. Phylogram to the left with branch lengths upscaled 10 times and outgroups removed. **Fig. S4. **Vouchers of selected samples specifically discussed in the text. **a **Sample EL123 *Isoetes hypsophila *Hand.-Mazz., Boufford 40096 (P), collected in China 2007; **b **Sample EL120 *?”Isoetes velata” *A.Braun, Vermulen et al. 1996-168 (L), collected in Portugal 1996; **c **Sample EL057 *Isoetes wormaldii *Sim, M. A. Pocock 20009 (BM), collected in a pond near Makhanda (previously known as Grahamstown), South Africa in 1955.

**Additional file 2. Appendix S1.** List of taxon names, distributions, DNA voucher information (including area and year of collection), lab identity numbers, and accession numbers for sequences used in the analyses. **Appendix S2.** Primer information.

## Data Availability

The data generated and analyzed during the current study are available in GenBank, GenBank Overview (nih.gov). The aligned datasets are available from the corresponding author on reasonable request.

## Abbreviation

Mya: Million years before present
