# Supplementary material for: Phylogeny of Merlin’s grass (Isoetaceae): revealing an “Amborella syndrome” and the importance of geographic distribution for understanding current and historical diversity
Source: BMC Ecol Evol. 2022 Mar 16;22:32. doi: 10.1186/s12862-022-01988-w (PMC8928685; doi:10.1186/s12862-022-01988-w)
Supplement: Supplementary file 1 — Additional file 1: Fig. S1. Maximum likelihood analysis of plastid data (ndhC-ndhK, rbcL, rpoC1, ycf1, ycf66, and trnVUAC and its subsequent spacer). Bootstrap support values and Bayesian posterior probabilities (as estimated in a separate analysis in MrBayes) are indicated on the tree as follows: maximum likelihood bootstrap (BS) / Bayesian posterior probability (PP). Clade names in green (A-E), subclade names in color following the scheme in Fig. 1 of the main text, and sample names in red are discussed in the text. Phylogram to the left with branch lengths upscaled 10 times and outgroups removed. Fig. S2. Maximum likelihood analysis of nuclear ribosomal data (nrITS). Bootstrap support values and Bayesian posterior probabilities (as estimated in a separate analysis in MrBayes) are indicated on the tree as follows: maximum likelihood bootstrap (BS) / Bayesian posterior probability (PP). Clade names in blue (A-E), subclade names in color following the scheme in Fig. 1 of the main text, and sample names in red are discussed in the text. Phylogram to the left. Fig. S3. Maximum likelihood analysis of plastid (ndhC-ndhK, rbcL, rpoC1, ycf1, ycf66, and trnVUAC and its subsequent spacer) and nuclear ribosomal data (nrITS). This analysis is equivalent to that depicted in Fig. 1 of the main text, with one exception: here, nuclear ribosomal data is also included for Isoetes wormaldii and eight outgroup taxa representing the Selaginellaceae and Lycopodiaceae. Despite potential difficulties to infer positional homology when aligning the nrITS sequences of outgroups and Isoetes wormaldii with those of the remaining Isoetes, most results are identical to those shown in Fig. 1. Bootstrap support values are indicated on the tree. Clade names in purple (A-E) and subclade names in color following the scheme in Fig. 1 of the main text, are discussed. Phylogram to the left with branch lengths upscaled 10 times and outgroups removed. Fig. S4. Vouchers of selected samples specifically di [file 12862_2022_1988_MOESM1_ESM.pdf]

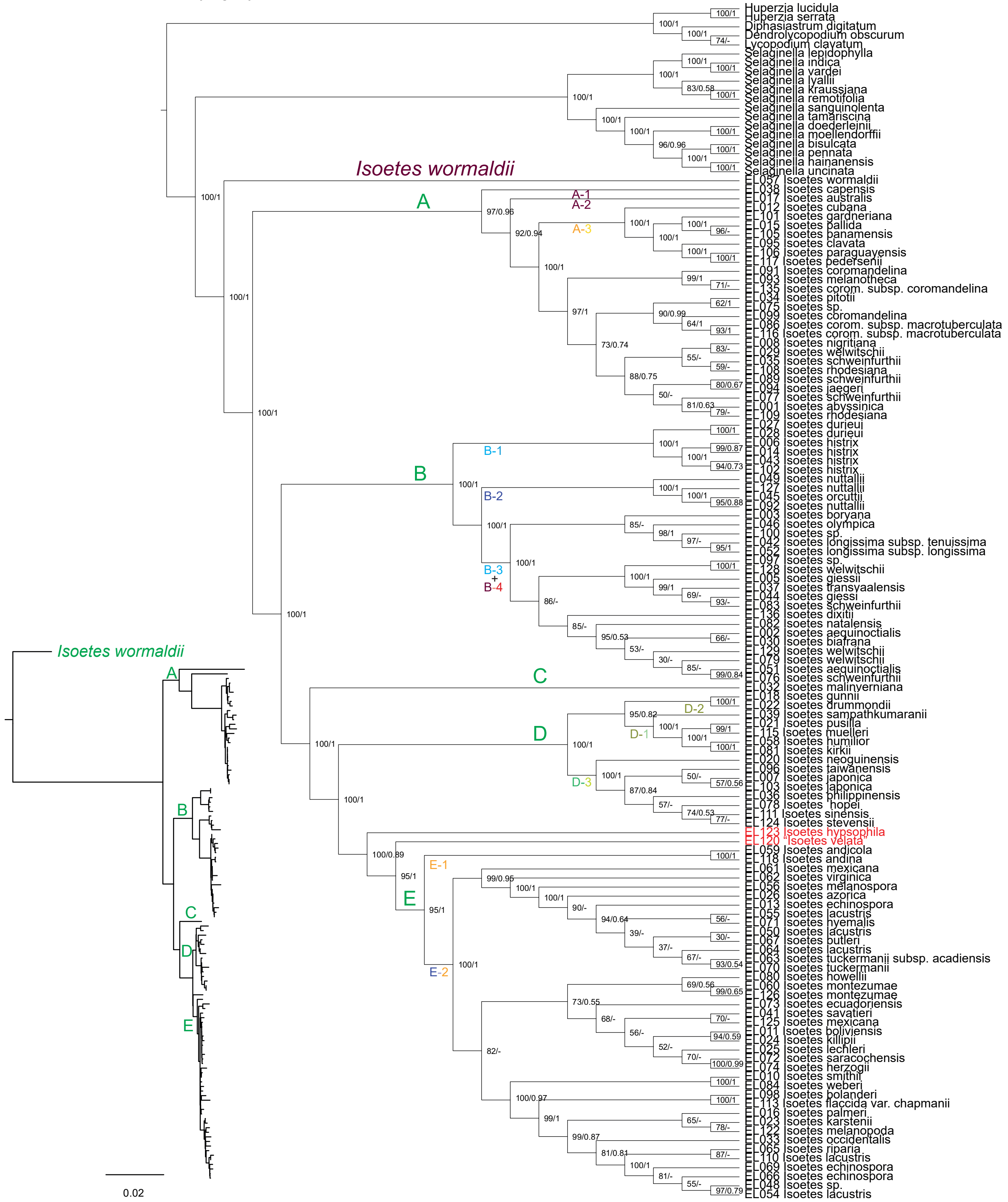

Fig. S1 Maximum likelihood analysis of plastid data (*ndhC-ndhK, rbcL, rpoC1, ycf1, ycf66*, and *trnV*UAC and its subsequent spacer). Bootstrap support values and Bayesian posterior probabilities (as estimated in a separate analysis in MrBayes) are indicated on the tree as follows: maximum likelihood bootstrap (BS) / Bayesian posterior probability (PP). Clade names in green (A-E), subclade names in color following the scheme in Fig. 1 of the main text, and sample names in red are discussed in the text. Phylogram to the left with branch lengths upscaled 10 times and outgroups removed.

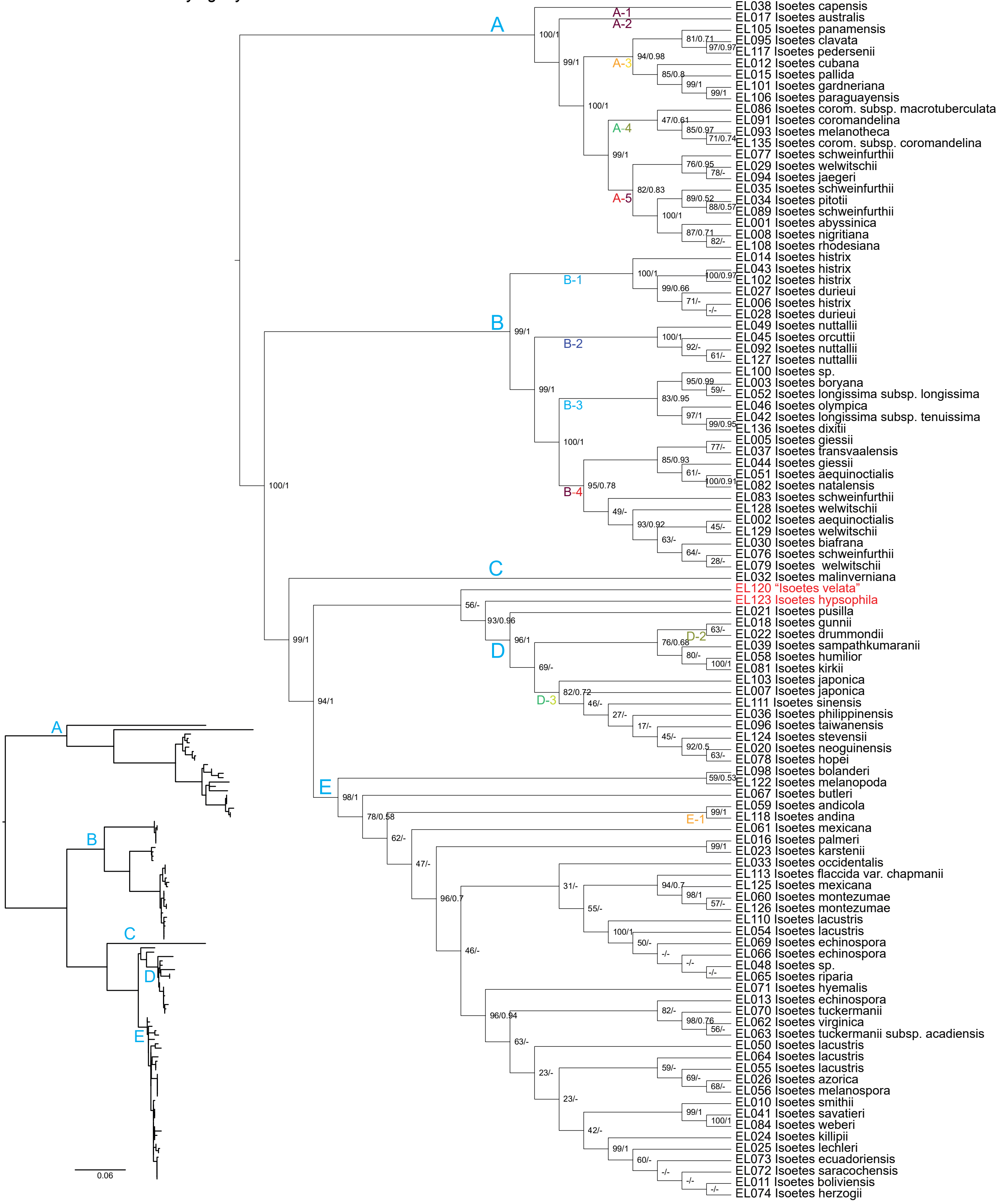

Fig. S2 Maximum likelihood analysis of nuclear ribosomal data (nrITS). Bootstrap support values and Bayesian posterior probabilities (as estimated in a separate analysis in MrBayes) are indicated on the tree as follows: maximum likelihood bootstrap (BS) / Bayesian posterior probability (PP). Clade names in blue (A-E), subclade names in color following the scheme in Fig. 1 of the main text, and sample names in red are discussed in the text. Phylogram to the left.

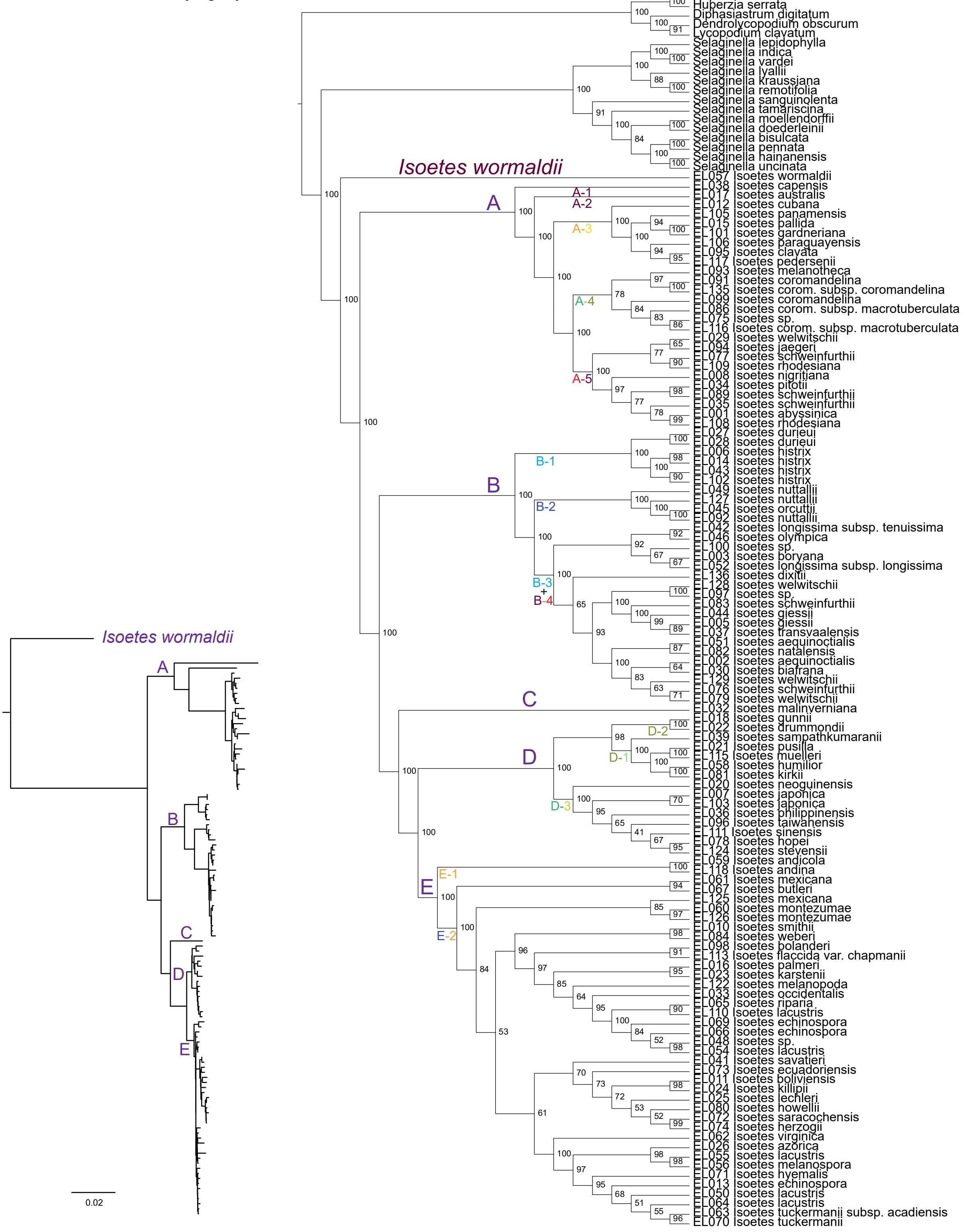

Fig. S3 Maximum likelihood analysis of plastid (*ndhC-ndhK, rbcL, rpoC1, ycf1, ycf66*, and *trnV*UAC and its subsequent spacer) and nuclear ribosomal data (nrITS). This analysis is equivalent to that depicted in Fig. 1 of the main text, with one exception: here, nuclear ribosomal data is also included for *Isoetes wormaldii* and eight outgroup taxa representing the Selaginellaceae and Lycopodiaceae. Despite potential difficulties to infer positional homology when aligning the nrITS sequences of outgroups and *Isoetes wormaldii* with those of the remaining *Isoetes*, most results are identical to those shown in Fig. 1. Bootstrap support values are indicated on the tree. Clade names in purple (A-E) and subclade names in color following the scheme in Fig. 1 of the main text, are discussed. Phylogram to the left with branch lengths upscaled 10 times and outgroups removed.

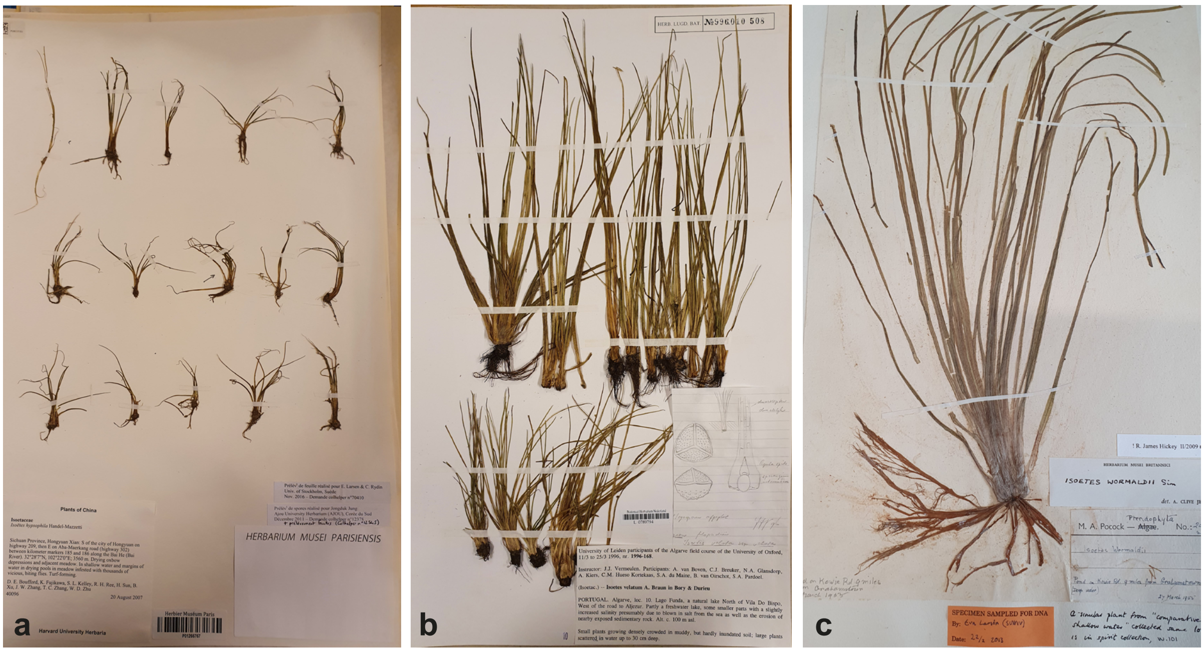

Fig. S4 Vouchers of selected samples specifically discussed in the text. **a** Sample EL123 *Isoetes hypsophila* Hand.-Mazz., Boufford 40096 (P), collected in China 2007; **b** Sample EL120 *?"Isoetes velata"* A.Braun, Vermulen et al. 1996-168 (L), collected in Portugal 1996; **c** Sample EL057 *Isoetes wormaldii* Sim, M. A. Pocock 20009 (BM), collected in a pond near Makhanda (previously known as Grahamstown), South Africa in 1955.
