# Supplementary material for: Phylogeny of Merlin’s grass (Isoetaceae): revealing an “Amborella syndrome” and the importance of geographic distribution for understanding current and historical diversity
Source: BMC Ecol Evol. 2022 Mar 16;22:32. doi: 10.1186/s12862-022-01988-w (PMC8928685; doi:10.1186/s12862-022-01988-w)
Supplement: Supplementary file 2 — Additional file 2. Appendix S1. List of taxon names, distributions, DNA voucher information (including area and year of collection), lab identity numbers, and accession numbers for sequences used in the analyses. Appendix S2. Primer information. [file 12862_2022_1988_MOESM2_ESM.pdf]

Appendix S1. List of taxon names, distributions, DNA voucher information (including area and year of collection), lab identity numbers, and accession numbers for sequences used in the analyses. References are given for sequences taken from GenBank. Taxon names and authority follow Troìa et al. [1] and Tropicos [2] with a few exceptions (see notes below). Distributions of *Isoetes* were extracted from Troìa et al. [1], Tropicos [2], GBIF.org [3], and SANBI's Red List of South African Plants [4]. Area names follow the World Geographical Scheme for Recording Plant Distributions [5]. An exception is the Mediterranean distribution, which refers to an occurrence in either one of the 22 sovereign countries in Europe, Africa and Temperate Asia that borders the Mediterranean Sea.

Lab. Ident.	Taxon	Nomenclatural notes	Distribution	DNA Voucher	Area/Year of collection	*ndhC-ndhK*	*rbcL*	*rpoC1*	*ycf1*	*ycf66*	*trnV*^{UAC}	nrITS
EL001	*Isoetes abyssinica* Chiov.		Northeastern Trop. Africa	Lewalle 5192 (BR)	Burundi 1971	-	OM691982*	OM870153*	OM869999*	OM691792*	-	OM719898*
EL002	*Isoetes aequinoctialis* Welw. ex A.Braun		Southern and Trop. Africa	Kornas 3453 (BR)	Zambia 1973	OM691715*	OM691983*	OM870154*	OM870000*	OM691793*	OM691883*	OM719899*
EL051	*Isoetes aequinoctialis* Welw. ex A.Braun		Southern and Trop. Africa	Giess 15279 (S)	Nambia 1978	OM691751*	OM692024*	OM870196, OM870292, OM870348*	OM870042*	OM691828*	OM691920*	OM719939*
EL059	*Isoetes andicola* (Amstutz) L.D.Gómez		Western South America	Marshall s.n. (BM)	Peru 1961	-	OM692031*	OM870203, OM870299, OM870355*	OM870049, OM870122*	OM691835*	OM691927*	OM719945*
EL118	*Isoetes andina* Spruce ex Hook.		Western & Northern South America	Larsen & Eriksen 19 (WU)	Ecuador 1985	OM691785*	OM692079*	OM870251, OM870382*	OM870097, OM870142*	OM691875*	OM691971*	OM719983*
EL017	*Isoetes australis* S.Williams		Western Australia	Orchard 1274 (MEL)	Australia 1968	OM691724*	OM691996*	OM870167*	OM870012*	OM691803*	OM691893*	OM719911*
EL026	*Isoetes azorica* Durieu ex Milde		The Azores	Gonalves 2611 (BM)	The Azores 1971	OM691731*	OM692004*	OM870175*	OM870020*	OM691810*	OM691899*	OM719919*
EL030	*Isoetes biafrana* Alston		Trop. Africa	Le Testu 3016 (BM)	C. Afr. Rep. 1951	OM691735*	OM692008*	OM870179*	OM870024*	OM691814*	OM691903*	OM719923*
EL098	*Isoetes bolanderi* Engelm.		Western Canada, Northwestern & Southwestern USA	Munz s.n. (C)	USA 1926	OM691775*	OM692064*	OM870235, OM870375*	OM870081*	OM691863*	OM691956*	OM719972*
EL011	*Isoetes boliviensis* U.Weber		Western South America	Hickey 753 (W)	Bolivia 1980	-	OM691990*	OM870161, OM870277, OM870334*	OM870006, OM870111*	-	-	OM719905*
EL003	*Isoetes boryana* Durieu		Southwestern Europe	Rouet 430 (BR)	France 1941	-	OM691984*	OM870155*	-	-	-	OM719900*
EL067	*Isoetes butleri* Engelm.		South-Central North America	Bush 7547 (BM)	USA 1915	-	OM692039*	OM870211*	OM870057*	-	OM691935*	OM719953*
EL038	*Isoetes capensis* A.V.Duthie		Southern Africa	Duthie s.n. (BM)	South Africa 1934	-	-	-	OM870031*	-	-	OM719929*
EL095	*Isoetes clavata* U.Weber		Northern South America	Boudrie 3922 (P)	French Guiana 2003	OM691774*	OM692061*	OM870233, OM870325, OM870374*	OM870079*	OM691862*	OM691954*	OM719971*
EL091	*Isoetes coromandelina* L.f.		Indian Subcontinent, Indo-China, Australia	Cook 5327 (P)	India 1995	OM691773*	OM692058*	OM870230, OM870322, OM870373*	OM870075*	OM691858*	OM691951*	OM719968*
EL099	*Isoetes coromandelina* L.f.		Indian Subcontinent, Indo-China, Australia	Larsen 8398 (C)	Thailand 1961	-	OM692065*	OM870236, OM870326*	OM870082, OM870137*	OM691864*	OM691957*	-
EL135	*Isoetes coromandelina* L.f. subsp. *coromandelina*	Det. *Isoetes indica* D.D.Pant & G.K.Srivast.	India	Srivastava 450268 (S)	India 2000	-	OM692089*	OM870261, OM870332*	OM870107*	-	-	OM719993*
EL086	*Isoetes coromandelina* L.f. subsp. *macro-tuberculata* C.R. Marsden		Australia (northern)	Walsh & Coles 4441 (MEL)	Australia 1996	OM691771*	OM692056*	OM870228, OM870320, OM870371*	OM870073, OM870133*	OM691856*	OM691949*	OM719966*
EL116	*Isoetes coromandelina* L.f. subsp. *macro-*		Australia (northern)	Fryxell & Carven 4249 (MEL)	Australia 1983	-	-	OM870249*	OM870095*	-	OM691969*	-

Lab. Ident.	Taxon	Nomenclatural notes	Distribution	DNA Voucher	Area/Year of collection	*ndhC-ndhK*	*rbcL*	*rpoC1*	*ycf1*	*ycf66*	$trnV^{UAC}$	nrITS
	tuberculata C.R. Marsden											
EL012	*Isoetes cubana* Engelm.		Caribbean, Central America, Mexico	Hickey & Hickey 979 (W)	Mexico 1986	OM691720*	OM691991*	OM870162*	OM870007*	OM691798*	OM691888*	OM719906*
EL136	*Isoetes dixitii* Shende		India	Patil 6 (K)	India 1973	-	OM692090*	OM870262*	OM870108, OM870145*	-	OM691981*	OM719994*
EL022	*Isoetes drummondii* A.Braun		Australia	Beauglehole 75018 (MEL)	Australia 1983	OM691728*	OM692000*	OM870171*	OM870016*	OM691807*	OM691896*	OM719915*
EL027	*Isoetes durieui* Bory		Mediterranean	Byfield s.n. (BM)	Turkey 1992	OM691732*	OM692005*	OM870176*	OM870021*	OM691811*	OM691900*	OM719920*
EL028	*Isoetes durieui* Bory		Mediterranean	De Retz 65141 (BM)	France 1972	OM691733*	OM692006*	OM870177*	OM870022*	OM691812*	OM691901*	OM719921*
EL013	*Isoetes echinospora* Durieu		Europe, Northern America, Asia-Temperate	Ford 609 (W)	Canada 2006	OM691721*	OM691992*	OM870163*	OM870008*	OM691799*	OM691889*	OM719907*
EL066	*Isoetes echinospora* Durieu	Det. *Isoetes muricata* Durieu	Europe, Northern America, Asia-Temperate	Rolland-Germain 2339 (BM)	Canada 1949	-	OM692038*	OM870210, OM870267, OM870305*	OM870056, OM870124*	OM691841*	OM691934*	OM719952*
EL069	*Isoetes echinospora* Durieu	Det. *Isoetes braunii* Durieu	Europe, Northern America, Asia-Temperate	Garton 1573 (BM)	Canada 1951	-	OM692040*	OM870212*	-	OM691842*	-	OM719954*
EL073	*Isoetes ecuadoriensis* Aspl.		Ecuador	Edwards, Farrar & Garrett 2102 (BM)	Ecuador 1969	OM691762*	OM692044*	OM870216, OM870309, OM870363*	OM870061, OM870126*	OM691846*	OM691939*	OM719957*
EL113	*Isoetes flaccida* var. *chapmanii* Engelm.		Southeastern USA	Godfrey 61963 (BR)	USA 1962	-	OM692076*	OM870247*	OM870093, OM870140*	OM691872*	OM691967*	OM719981*
EL101	*Isoetes gardneriana* Kunze ex A.Braun		Brazil, Southern South America	Pedersen 19654 (C)	Argentina 1984	OM691777*	OM692067*	OM870238, OM870327*	OM870084*	OM691866*	OM691959*	OM719974*
EL044	*Isoetes giessi* Launert		Namibia	Giess, Volk & Bleissner 5564 (S)	Nambia 1963	OM691745*	OM692018*	OM870190, OM870286, OM870343*	OM870036*	OM691823*	OM691914*	OM719934*
EL005	*Isoetes giessii* Launert		Namibia	Giess, Volk & Bleissner 5564 (BR)	Namibia 1963	OM691716*	OM691985*	OM870156*	OM870001*	OM691794*	OM691884*	OM719901*
EL018	*Isoetes gunnii* A.Braun		Tasmania	Forbes 1270 (MEL)	Australia 1983	OM691725*	OM691997*	OM870168, OM870280, OM870337*	OM870013, OM870113*	OM691804*	-	OM719912*
EL074	*Isoetes herzogii* U.Weber		Bolivia	Halls s.n. (BM)	Bolivia 1984	OM691763*	OM692045*	OM870217, OM870310, OM870364*	OM870062*	OM691847*	OM691940*	OM719958*
EL006	*Isoetes histrix* Bory		Mediterranean	Iberite 15886 (BR)	Italy 1991	OM691717*	OM691986*	OM870157, OM870276, OM870333*	OM870002, OM870109, OM870146*	OM691795*	OM691885*	OM719902*
EL014	*Isoetes histrix* Bory		Mediterranean	Aedo et al s.n. (W)	Bulgaria 2004	OM691722*	OM691993*	OM870164, OM870278, OM870335*	OM870009*	OM691800*	OM691890*	OM719908*
EL043	*Isoetes histrix* Bory	Det. *Isoetes velata* A.Braun subsp. *adspersa* (A.Braun) Batt. & Trab.	Mediterranean	Podlech 53922 (W)	Morocco 1997	OM691744*	OM692017*	OM870189, OM870285, OM870342*	OM870035, OM870116, OM870148*	OM691822*	OM691913*	OM719933*
EL102	*Isoetes histrix* Bory		Mediterranean	Lewalle 8610 (C)	Morocco 1977	OM691778*	OM692068*	OM870239, OM870328*	OM870085, OM870138*	-	OM691960*	OM719975*
EL078	*Isoetes hopei* J.R.Croft		Papuasia	Hope s.n. (BM)	Indonesia	OM691766*	OM692049*	OM870221,	OM870066*	OM691851*	OM691944*	OM719961*

Lab. Ident.	Taxon	Nomenclatural notes	Distribution	DNA Voucher	Area/Year of collection	*ndhC-ndhK*	*rbcL*	*rpoC1*	*ycf1*	*ycf66*	$trnV^{UAC}$	nrITS
					1984			OM870314, OM870367*				
EL080	*Isoetes howellii* Engelm.		Northwestern & Southwestern USA & Western Canada	Thorne & Lathrop 37942 (BM)	USA 1969	OM691768*	OM692051*	OM870223, OM870270, OM870316*	OM870068, OM870129*	-	-	-
EL058	*Isoetes humilior* F.Muell. ex A.Braun[a]		Australia	Darbyshire 134 (BM)	Australia 1961	OM691755*	OM692030*	OM870202, OM870266, OM870298, OM870354*	OM870048, OM870121*	OM691834*	OM691926*	OM719944*
EL071	*Isoetes hyemalis* D.F.Brunt		Southeastern USA	Brunton & McIntosh 12560 (BM)	USA 1996	OM691760*	OM692042*	OM870214, OM870307, OM870362*	OM870059*	OM691844*	OM691937*	OM719999, OM719885*
EL123	*Isoetes hypsophila* Hand.-Mazz.		China	Boufford 40096 (P)	China 2007	-	OM692082*	OM870254*	OM870100*	-	OM691974*	OM719986*
EL094	*Isoetes jaegeri* Pitot		Trop. Africa	Jaeger 5236 (P)	Tanzania 1958	-	OM692060*	OM870232, OM870275, OM870324*	OM870078, OM870135*	OM691861*	OM691953*	OM719970*
EL007	*Isoetes japonica* A.Braun		Eastern Asia	Makino 30547 (BR)	Japan 1904	-	OM691987*	OM870158*	OM870003, OM870110*	-	-	OM719996, OM719881*
EL103	*Isoetes japonica* A.Braun		Eastern Asia	Amano 328 (C)	Japan 1986	OM691779*	OM692069*	OM870240, OM870329, OM870377*	OM870086*	OM691867*	OM691961*	OM720001, OM719889*
EL023	*Isoetes karstenii* A.Braun		Northern & Western South America	Huber s.n. (BM)	Venezuela 1971	OM691729*	OM692001*	OM870172, OM870281, OM870338*	OM870017*	OM691808*	-	OM719916*
EL024	*Isoetes killipii* C.V.Morton		Northern & Western South America	Grubb & Guymer P. 37 (BM)	Colombia 1957	-	OM692002*	OM870173, OM870282, OM870339*	OM870018*	-	OM691897*	OM719917*
EL081	*Isoetes kirkii* A.Braun		New Zealand	Chinnock P447 (BM)	New Zealand 1972	OM691769*	OM692052*	OM870224, OM870271, OM870317, OM870369*	OM870069, OM870130*	OM691853*	OM691946*	OM719963*
EL050	*Isoetes lacustris* L.		Europe, Northern America, Asia-Temperate	Jacobsen s.n. (S)	Greenland 1970	OM691750*	OM692023*	OM870195, OM870291, OM870347*	OM870041*	OM691827*	OM691919*	OM719938*
EL054	*Isoetes lacustris* L.	Det. *Isoetes setacea* Lam.	Europe, Northern America, Asia-Temperate	Øllgaard & Pedersen 180 (BM)	Denmark 1954	-	OM692026*	OM870198, OM870294, OM870350*	OM870044, OM870119*	OM691830*	OM691922*	OM719941*
EL055	*Isoetes lacustris* L.		Europe, Northern America, Asia-Temperate	Callé s.n. (BM)	France 1967	OM691753*	OM692027*	OM870199, OM870264, OM870295, OM870351*	OM870045, OM870120*	OM691831*	OM691923*	OM719998, OM719884*
EL064	*Isoetes lacustris* L.		Europe, Northern America, Asia-Temperate	Taylor 4904 (BM)	USA 1983	OM691759*	OM692036*	OM870208, OM870304, OM870359*	OM870054*	OM691840*	OM691932*	OM719950*
EL110	*Isoetes lacustris* L.	Det. *Isoetes setacea* Lam.	Europe, Northern America, Asia-Temperate	Tihimorov, Prokopova & Samarina 11060 (C)	Russia 1976	OM691783*	OM692074*	OM870245*	OM870091*	OM691871*	OM691966*	OM719979*
EL025	*Isoetes lechleri* Mett.		Western South America	Fernandez-Casas &	Bolivia 1982	OM691730*	OM692003*	OM870174*	OM870019*	OM691809*	OM691898*	OM719918*

Lab. Ident.	Taxon	Nomenclatural notes	Distribution	DNA Voucher	Area/Year of collection	*ndhC-ndhK*	*rbcL*	*rpoC1*	*ycf1*	*ycf66*	$trnV^{UAC}$	nrITS
				Molero 6619 (BM)								
EL052	*Isoetes longissima* Bory subsp. *longissima*	Det. *Isoetes velata* A.Braun subsp. *velata*	Mediterranean	De Retz 65107 (BR)	France 1972	OM691752*	OM692025*	OM870197, OM870293, OM870349*	OM870043*	OM691829*	OM691921*	OM719940*
EL042	*Isoetes longissima* Bory subsp. *tenuissima* (Boreau) Troía & Greuter	Det. *Isoetes tenuissima* Boreau	Mediterranean	Kowallik s.n. (W)	Italy 1963	OM691743*	OM692016*	OM870188, OM870284, OM870341*	OM870034, OM870115, OM870147*	-	OM691912*	OM719932*
EL032	*Isoetes malinverniana* Ces & De Not.		Italy	Raynal 20885 (BR)	Italy 1978	OM691736*	OM692009*	OM870180*	OM870025*	OM691815*	OM691904*	OM719924*
EL122	*Isoetes melanopoda* J.Gay & Durieu		Northcentral, Northeastern & Southeastern USA	Thomas 88386 (L)	USA 1984	OM691787*	OM692081*	OM870253, OM870384*	OM870099*	-	OM691973*	OM719985*
EL056	*Isoetes melanospora* Engelm.		Southeastern USA	Spongberg & Boufford 1726 (BM)	USA 1982	OM691754*	OM692028*	OM870200, OM870296, OM870352*	OM870046*	OM691832*	OM691924*	OM719942*
EL093	*Isoetes melanotheca* Alston		West Tropical Africa	Raynal 7693 (P)	Senegal 1961	-	-	-	OM870077*	OM691860*	-	OM719887*
EL061	*Isoetes mexicana* Underw.		Mexico	Pringle 8796 (BM)	Mexico 1904	-	OM692033*	OM870205, OM870301*	OM870051, OM870123, OM870149*	OM691837*	OM691929*	OM719947*
EL125	*Isoetes mexicana* Underw.		Mexico	Rzedowski 50430 (NY)	Mexico 1990	OM691788*	OM692084*	OM870256, OM870386*	OM870102, OM870144*	OM691878*	OM691976*	OM719988*
EL060	*Isoetes montezumae* A.A.Eaton		Mexico	McVaugh 13650 (BM)	Mexico 1952	OM691756*	OM692032*	OM870204, OM870300, OM870356*	OM870050*	OM691836*	OM691928*	OM719946*
EL126	*Isoetes montezumae* A.A.Eaton		Mexico	Hickey 940 (NY)	Mexico 1986	-	OM692085*	OM870257, OM870387*	OM870103*	OM691879*	OM691977*	OM719989*
EL115	*Isoetes muelleri* A.Braun		Australia	Stajsic 908 (MEL)	Australia 1993	-	OM692077*	OM870248, OM870331*	OM870094, OM870141*	OM691873*	OM691968*	-
EL082	*Isoetes natalensis* Baker	Accepted name: *Isoetes welwitschii* A.Br. ex Kuhn[d]	Madagascar, Southern Africa	Burrows 3734 (BM)	Namibia 1987	-	OM692053*	OM870225, OM870272*	OM870070, OM870131*	-	OM691947*	OM719964*
EL020	*Isoetes neoguineensis* Baker		Papua New Guinea	Craven 2717 (MEL)	Papua New Guinea 1974	OM691726*	OM691998*	OM870169*	OM870014*	OM691805*	OM691894*	OM719913*
EL008	*Isoetes nigritiana* A.Braun		West-Central Trop. Africa	De Wilde & De Wilde-Duyfjes 3518 (BR)	Cameroon 1964	OM691718*	OM691988*	OM870159*	OM870004*	OM691796*	OM691886*	OM719903*
EL092	*Isoetes nuttallii* A.Braun		Western Canada, Northwestern & Southwestern USA	Rose 67129 (P)	USA 1967	-	OM692059*	OM870231, OM870274, OM870323*	OM870076, OM870134, OM870150*	OM691859*	OM691952*	OM719969*
EL127	*Isoetes nuttallii* A.Braun		Western Canada, Northwestern & Southwestern USA	Halse 6485 (NY)	USA 2004	OM691789*	OM692086*	OM870258, OM870388*	OM870104*	OM691880*	OM691978*	OM719990*
EL049	*Isoetes nuttallii* A.Braun ex Engelm.		Western Canada, Northwestern & Southwestern USA	Macoun 86378 (S)	Canada 1908	OM691749*	OM692022*	OM870194, OM870290*	OM870040, OM870118*	-	OM691918*	OM719997, OM719883*
EL033	*Isoetes occidentalis* L.F.Hend.		Subarctic America; Western Canada; Northwestern USA;	Oettinger & Thorne 1268 (BM)	USA 1969	OM691737*	OM692010*	OM870181*	OM870026*	OM691816*	OM691905*	OM719925*

Lab. Ident.	Taxon	Nomenclatural notes	Distribution	DNA Voucher	Area/Year of collection	*ndhC-ndhK*	*rbcL*	*rpoC1*	*ycf1*	*ycf66*	*trnV*^{UAC}	nrITS
			Southwestern USA									
EL046	*Isoetes olympica* A.Braun		Syria, Turkey	Samuelsson 4566 (S)	Syria 1933	OM691747*	OM692020*	OM870192, OM870288, OM870345*	OM870038, OM870117*	OM691825*	OM691916*	OM719936*
EL045	*Isoetes orcuttii* A.A.Eaton		Southwestern USA, Mexico	Carter 471 (S)	USA 1934	OM691746*	OM692019*	OM870191, OM870287, OM870344*	OM870037*	OM691824*	OM691915*	OM719935*
EL015	*Isoetes pallida* Hickey		Mexico	Hickey & Hickey 962 (W)	Mexico 1986	OM691723*	OM691994*	OM870165*	OM870010*	OM691801*	OM691891*	OM719909*
EL016	*Isoetes palmeri* H.P.Fuchs		Northern & Western South America	Small 155 (W)	Colombia 1993	-	OM691995*	OM870166, OM870279, OM870336*	OM870011, OM870112*	OM691802*	OM691892*	OM719910*
EL105	*Isoetes panamensis* Maxon & C.V.Morton		Central America, Western South America, Brazil	Irwin, Harley & Smith 31615 (C)	Brazil 1971	-	OM692070*	OM870241, OM870378*	OM870087, OM870139, OM870152*	OM691868*	OM691962*	OM719976*
EL106	*Isoetes paraguayensis* nomen nudum		---	Pedersen 7624 (C)	Paraguay 1965	OM691780*	OM692071*	OM870242*	OM870088*	OM691869*	OM691963*	OM719977*
EL117	*Isoetes pedersenii* H.P.Fuchs ex E.I.Meza & Macluf		Western & Southern South America, Brazil	Abbott 16374 (WU)	Bolivia 1995	OM691784*	OM692078*	OM870250, OM870381*	OM870096*	OM691874*	OM691970*	OM719982*
EL036	*Isoetes philippinensis* Merr. & L.M.Perry		Malesia	Price 500 (BM)	Philippines 1969	OM691739*	OM692012*	OM870184*	OM870029*	OM691818*	OM691908*	OM719927*
EL034	*Isoetes pitotii* Alston		West Tropical Africa	Hall 3696 (BM)	Ghana 1967	-	-	OM870182*	OM870027, OM870114*	-	OM691906*	OM719882*
EL021	*Isoetes pusilla* C.R.Marsden & Chinnock		New South Wales; Victoria	Willis s.n. (MEL)	Australia 1981	OM691727*	OM691999*	OM870170*	OM870015*	OM691806*	OM691895*	OM719914*
EL108	*Isoetes rhodesiana* Alston	Accepted name: *Isoetes schweinfurthii* A.Braun ex Baker[d]	East Tropical Africa	Bidgood, Abdallah & Vollesen 1919 (C)	Tanzania 1991	OM691781*	OM692072*	OM870243, OM870379*	OM870089*	-	OM691964*	OM719978*
EL109	*Isoetes rhodesiana* Alston	Accepted name: *Isoetes schweinfurthii* A.Braun ex Baker[d]	East Tropical Africa	Vollesen 4586 (C)	Tanzania 1977	OM691782*	OM692073*	OM870244, OM870380*	OM870090*	OM691870*	OM691965*	-
EL065	*Isoetes riparia* Engelm. ex A.Braun		Eastern Canada, Northeastern & Southeastern USA	Jermy 12487 (BM)	Canada 1975	-	OM692037*	OM870209, OM870360*	OM870055*	-	OM691933*	OM719951*
EL039	*Isoetes sampathkumaranii* L.N.Rao[b]		Indian Subcontinent	Goswami s.n. (BM)	India (unknown year)	OM691741*	OM692014*	OM870186*	OM870032*	OM691820*	OM691910*	OM719930*
EL072	*Isoetes saracochensis* Hickey		Peru	Tutin 1406 (BM)	Peru 1937	OM691761*	OM692043*	OM870215, OM870269, OM870308*	OM870060, OM870125*	OM691845*	OM691938*	OM719956*
EL041	*Isoetes savatieri* Franch.		Southern South America	Weigend et al. 6930 (W)	Argentina 2002	OM691742*	OM692015*	OM870187, OM870283, OM870340*	OM870033*	OM691821*	OM691911*	OM719931*
EL076	*Isoetes schweinfurthii* A.Braun		Southern and Trop. Africa	Gilbert 878 (BM)	Ethiopia 1975	OM691764*	OM692047*	OM870219, OM870312, OM870365*	OM870064*	OM691849*	OM691942*	OM719959*
EL077	*Isoetes schweinfurthii*		Southern and Trop.	Garrod & Sanusi 6309	Nigeria 1977	OM691765*	OM692048*	OM870220,	OM870065,	OM691850*	OM691943*	OM719960*

Lab. Ident.	Taxon	Nomenclatural notes	Distribution	DNA Voucher	Area/Year of collection	*ndhC-ndhK*	*rbcL*	*rpoC1*	*ycf1*	*ycf66*	*trnV*^{UAC}	nrITS
	A.Braun		Africa	(BM)				OM870313, OM870366*	OM870128*			
EL083	*Isoetes schweinfurthii* A.Braun		Southern and Trop. Africa	Roux 1070 (BM)	South Africa 1982	OM691770*	OM692054*	OM870226, OM870273, OM870318*	OM870071*	OM691854*	-	OM720000, OM719886*
EL089	*Isoetes schweinfurthii* A.Braun		Southern and Trop. Africa	Bidgood, Leliyo & Vollesen 7304 (P)	Tanzania 2008	OM691772*	OM692057*	OM870229, OM870321, OM870372*	OM870074*	OM691857*	OM691950*	OM719967*
EL035	*Isoetes schweinfurthii* A.Braun.	Det. *Isoetes kersii* Wannt.	Southern and Trop. Africa	Kers 3130 (BM)	Namibia 1968	OM691738*	OM692011*	OM870183*	OM870028*	OM691817*	OM691907*	OM719926*
EL111	*Isoetes sinensis* T.C.Palmer		China	[Illegible] s.n. (C)	China 1927	-	OM692075*	OM870246, OM870330*	OM870092*	-	-	OM719980*
EL010	*Isoetes smithii* H.P.Fuchs		Brazil	Callé 95840 (BR)	Brazil 1935	OM691719*	OM691989*	OM870160*	OM870005*	OM691797*	OM691887*	OM719904*
EL048	*Isoetes* sp.			Suksdorf s.n. (S)	USA 1909	OM691748*	OM692021*	OM870193, OM870263, OM870289, OM870346*	OM870039*	OM691826*	OM691917*	OM719937*
EL075	*Isoetes* sp.			Wallace 5915 (BM)	Myanmar 1945	-	OM692046*	OM870218, OM870311*	OM870063, OM870127*	OM691848*	OM691941*	-
EL097	*Isoetes* sp.			Rajaonary, Ravololomanana & Porembski 205 (S)	Madacascar	-	OM692063*	OM870234*	OM870080, OM870136, OM870151*	-	OM691955*	-
EL100	*Isoetes* sp.	Det. *Isoetes delilei* (Bory) Rothm.	Western Mediterranean	Ortiz & Pueche 1469 (C)	Spain 1977	OM691776*	OM692066*	OM870237, OM870376*	OM870083*	OM691865*	OM691958*	OM719973*
EL124	*Isoetes stevensii* J.R.Croft		Papua New Guinea	Schodde 1843 (C)	Papua New Guinea 1961	-	OM692083*	OM870255, OM870385*	OM870101, OM870143*	OM691877*	OM691975*	OM719987*
EL096	*Isoetes taiwanensis* De Vol		China (Taiwan)	Knapp 1 (P)	Taiwan 2000	-	OM692062*	-	-	-	-	OM719888*
EL037	*Isoetes transvaalensis* C.Jermy & Schelpe		Southern Africa	Hilliard & Burtt 5989 (BM)	South Africa 1969	OM691740*	OM692013*	OM870185*	OM870030*	OM691819*	OM691909*	OM719928*
EL070	*Isoetes tuckermanii* A.Braun ex Engelm.		Eastern Canada, Northeastern USA	Brunton 10076 (BM)	Canada 1990	-	OM692041*	OM870213, OM870268, OM870306, OM870361*	OM870058*	OM691843*	OM691936*	OM719955*
EL063	*Isoetes tuckermanii* A.Braun ex Engelm. subsp. *acadiensis* (Kott) D.F.Brunt.	Det. *Isoetes acadiensis* Kott	Eastern Canada, Northeastern USA	Brunton 10126 (BM)	Canada 1990	OM691758*	OM692035*	OM870207, OM870303, OM870358*	OM870053*	OM691839*	OM691931*	OM719949*
EL120	*?Isoetes velata* A.Braun[c]	Accepted name: *Isoetes longissima* Bory subsp. *longissima*	Mediterranean	Vermeulen et al. 1996-168 (L)	Portugal 1996	OM691786*	OM692080*	OM870252, OM870383*	OM870098*	OM691876*	OM691972*	OM719984*
EL062	*Isoetes virginica* N.Pfeiff.	Det.: *Isoetes piedmontana* (N.Pheiff.) C.F.Reed	South-central & Southeastern USA	Spongberg 1743 (BM)	USA 1982	OM691757*	OM692034*	OM870206, OM870302, OM870357*	OM870052*	OM691838*	OM691930*	OM719948*
EL084	*Isoetes weberi* Herter		Brazil	Rambo 42698 (BM)	Brazil 1949	-	OM692055*	OM870227, OM870319, OM870370*	OM870072, OM870132*	OM691855*	OM691948*	OM719965*
EL029	*Isoetes welwitschii* A.Braun		Southern and Trop. Africa	Wingfield 2032 (BM)	Tanzania 1972	OM691734*	OM692007*	OM870178*	OM870023*	OM691813*	OM691902*	OM719922*

**Lab. Ident.**	**Taxon**	**Nomenclatural notes**	**Distribution**	**DNA Voucher**	**Area/Year of collection**	***ndhC-ndhK***	***rbcL***	***rpoC1***	***ycf1***	***ycf66***	***trnV^UAC^***	**nrITS**
EL079	*Isoetes welwitschii* A.Braun		Southern and Trop. Africa	Gilbert & Thulin 863 (BM)	Ethiopia 1975	OM691767*	OM692050*	OM870222, OM870315, OM870368*	OM870067*	OM691852*	OM691945*	OM719962*
EL128	*Isoetes welwitschii* A.Braun		Southern and Trop. Africa	Razafimandimbison, Razafindrahaja, Atalahy & Swenson 2142 (S)	Madagascar 2018	OM691790*	OM692087*	OM870259*	OM870105*	OM691881*	OM691979*	OM719991*
EL129	*Isoetes welwitschii* A.Braun		Southern and Trop. Africa	Razafimandimbison, Razafindrahaja, Atalahy & Swenson 2151 (S)	Madagascar 2018	OM691791*	OM692088*	OM870260*	OM870106*	OM691882*	OM691980*	OM719992*
EL057	*Isoetes wormaldii* Sim.		South Africa (Eastern Cape)	Pocock 20009 (BM)	South Africa 1955	-	OM692029*	OM870201, OM870265, OM870297, OM870353*	OM870047*	OM691833*	OM691925*	(OM719943*)
GB	*Dendrolycopodium obscurum* (L.) A.Haines		---	---	---	MH549637[6]	MH549637[6]	MH549637[6]	MH549637[6]	MH549637[6]	MH549637[6]	-
GB	*Diphasiastrum digitatum* (Dill. ex A.Braun) Holub		---	---	---	MH549638[6]	MH549638[6]	MH549638[6]	MH549638[6]	MH549638[6]	MH549638[6]	-
GB	*Huperzia lucidula* (Michx.) Trevis.		---	---	---	AY660566[7]	AY660566[7]	AY660566[7]	AY660566[7]	AY660566[7]	AY660566[7]	(KF977440[8])
GB	*Huperzia serrata* (Thunb.) Trevis.		---	---	---	KX426071[9]	KX426071[9]	KX426071[9]	KX426071[9]	KX426071[9]	KX426071[9]	(DQ234271[10])
GB	*Lycopodium clavatum* L.		---	---	---	MH549642[6]	MH549642[6]	MH549642[6]	MH549642[6]	MH549642[6]	MH549642[6]	-
GB	*Selaginella bisulcata* Spring		---	---	---	MH598531[11]	MH598531[11]	MH598531[11]	MH598531[11]	-	-	-
GB	*Selaginella doederleinii* Hieron.		---	---	---	MH598532[11]	MH598532[11]	MH598532[11]	MH598532[11]	-	-	-
GB	*Selaginella hainanensis* X.C.Zhang & Noot.		---	---	---	-	MH598533[11]	MH598533[11]	MH598533[11]	-	-	-
GB	*Selaginella indica* (Milde) R.M.Tryon		---	---	---	-	MK156801[12]	MK156801[12]	MK156801[12]	-	-	(AF419020[13])
GB	*Selaginella kraussiana* (Kunze) A.Braun		---	---	---	MH549643[6]	MH549643[6]	MH549643[6]	MH549643[6]	-	-	(KT161746[14])
GB	*Selaginella lepidophylla* (Hook. & Grev.) Spring		---	---	---	-	MK089531[6]	MK089531[6]	MK089531[6]	-	-	(AF419001[13])
GB	*Selaginella lyallii* (Hook. & Grev.) Spring		---	---	---	-	MK156800[11]	MK156800[11]	MK156800[11]	-	-	-
GB	*Selaginella moellendorffii* Hieron.		---	---	---	MG272484[11]	MG272484[11]	MG272484[11]	MG272484[11]	-	-	(KT161774[14])
GB	*Selaginella pennata* (D.Don) Spring		---	---	---	MH598534[11]	MH598534[11]	MH598534[11]	MH598534[11]	-	-	-
GB	*Selaginella remotifolia* Spring		---	---	---	MH598535[11]	MH598535[11]	MH598535[11]	MH598535[11]	-	-	(KT161813[14])
GB	*Selaginella sanguinolenta* (L.) Spring		---	---	---	-	MH598536[11]	MH598536[11]	MH598536[11]	-	-	(KT161823[14])
GB	*Selaginella tamariscina* (P.Beauv.) Spring		---	---	---	-	MH598537[11]	MH598537[11]	MH598537[11]	-	-	-
GB	*Selaginella uncinata* (Desv.) Spring		---	---	---	MG272483[11]	MG272483[11]	MG272483[11]	MG272483[11]	-	-	-

Lab. Ident.	Taxon	Nomenclatural notes	Distribution	DNA Voucher	Area/Year of collection	*ndhC-ndhK*	*rbcL*	*rpoC1*	*ycf1*	*ycf66*	*trnV*[UAC]	nrITS
GB	*Selaginella vardei* H.Lév.		---	---	---	-	MG272482[12]	MG272482[12]	MG272482[12]	-	-	-

Notes: *(asterisk) denotes sequences newly produced for the present study. Asterisk or accession number of nrITS sequences within parenthesis denote sequences that were included only in the analysis presented in Additional file 1: Fig. S3; otherwise excluded from all analyses due to potential problems with inference of positional homology.

Nomenclatural remarks

a. The authority of *Isoetes humilior* is unclear; it is variably stated as F.Muell. [1] or A.Braun [2, 15]. The species is referred to as *Isoetes humilior* F.Muell. in the original description [16] and we have therefore chosen to use F.Muell. ex A.Braun.
b. Spelling of *Isoetes sampathkumaranii* follows Singh et al. [17].
c. It is unclear what species this sample represents. Indicatively, we use the name on the herbarium sheet: *Isoetes velata* A.Braun.
d. Name is used even though it is currently considered a synonym, because the respective accepted names *Isoetes schweinfurthii* A.Braun and *I. welwitschii* A.Braun are clearly not tenable.

References

1. Troìa A, Pereira JB, Kim C, Taylor WC. The genus *Isoetes* (Isoetaceae): a provisional checklist of the accepted and unresolved taxa. Phytotaxa. 2016;277(2):101-45.
2. Tropicos. Missouri Botanical Garden. 2021. http://www.tropicos.org. Accessed 27 Apr 2021.
3. GBIF.org. Home Page. 2021. https://www.gbif.org. Accessed 29 Jan 2021.
4. Victor JE, Dold AP. *Isoetes wormaldii* Sim. 2007. http://redlist.sanbi.org/. Accessed 3 Feb 2021.
5. Brummitt RK: World geographic scheme for recording plant distributions, 2nd ed. Carnegie Mellon University, Pittsburgh: Hunt Institute for Botanical Documentation; 2001.
6. Mower JP, Ma P-F, Grewe F, Taylor A, Michael TP, VanBuren R et al. Lycophyte plastid genomics: extreme variation in GC, gene and intron content and multiple inversions between a direct and inverted orientation of the rRNA repeat. New Phytol. 2019;222(2):1061-75.
7. Wolf PG, Karol KG, Mandoli DF, Kuehl J, Arumuganathan K, Ellis MW et al. The first complete chloroplast genome sequence of a lycophyte, *Huperzia lucidula* (Lycopodiaceae). Gene. 2005;350(2):117-28.
8. Begley-Miller DR, Hipp AL, Brown BH, Hahn M, Rooney TP. White-tailed deer are a biotic filter during community assembly, reducing species and phylogenetic diversity. AoB Plants. 2014;6:plu030.
9. Guo Z-Y, Zhang H-R, Shrestha N, Zhang X-C. Complete chloroplast genome of a valuable medicinal plant, *Huperzia serrata* (Lycopodiaceae), and comparison with its congener. Appl Plant Sci. 2016;4(11):1600071.
10. Jiang J, Liu Q, Zhang X, Gong Y, Qian L, Bao K et al. Direct submission.
11. Zhang H-R, Xiang Q-P, Zhang X-C. The unique evolutionary trajectory and dynamic conformations of DR and IR/DR-coexisting plastomes of the early vascular plant Selaginellaceae (Lycophyte). Genome Biol Evol. 2019;11(4):1258-74.
12. Zhang H-R, Zhang X-C, Xiang Q-P. Directed repeats co-occur with few short-dispersed repeats in plastid genome of a spikemoss, *Selaginella vardei* (Selaginellaceae, Lycopodiopsida). BMC Genomics. 2019;20(1):484.
13. Arrigo N, Therrien J, Anderson CL, Windham MD, Haufler CH, Barker MS. A total evidence approach to understanding phylogenetic relationships and ecological diversity in *Selaginella* subg. *Tetragonostachys*. Am J Bot. 2013;100(8):1672-82.
14. Zhou X-M, Rothfels CJ, Zhang L, He Z-R, Le Péchon T, He H et al. A large-scale phylogeny of the lycophyte genus *Selaginella* (Selaginellaceae: Lycopodiopsida) based on plastid and nuclear loci. Cladistics. 2016;32(4):360-89.
15. IPNI. International Plant Names Index, Published on the Internet http://www.ipni.org, The Royal Botanic Gardens, Kew, Harvard University Herbaria & Libraries and Australian National Botanic Gardens. 2021. Accessed 27 Jan 2021.
16. Braun A. Plantae Muellerianae - Isoeteae. Linnaea. 1853;25:722.
17. Singh SK, Shukla PK, Brunton DF, Dubey NK, Shukla SK. The taxonomy and conservation status of *Isoetes* (Isoetaceae; Lycopodiopsida) in India. Bot Lett. 2021;168(2):200-26.

Appendix S2 Primer information

Region	Primer	Sequence (5'--3')	Reference
**nrITS**			
	18SF-ITS-u1F	GGA AGK ARA AGT CGT AAC AAG G	Cheng et al. [1]
	5.8SF-ITS-u3F	CAW CGA TGA AGA ACG YAG C	Cheng et al. [1]
	5.8SR-ITS-u2R	GCG TTC AAA GAY TCG ATG RTT C	Cheng et al. [1]
	26SR-ITS-u4R	RGT TTC TTT TCC TCC GCT TA	Cheng et al. [1]
	ITS-5R	CCT CCG CTT AAT GAT ATG C	The present study
ndhC-			
ndhK	trnV-1785F	GCG AAA CAA TTA CAC TTG C	The present study
	trnV-3282R	CGG CCC TAC ATT TCA TTA TCT G	The present study
rbcL			
	rbcL-1F	CTC CTG ATT ATA AGA CCA AAG ACA CCG	Larsén and Rydin [2]
	rbcL-565F	GTT TAT GCG TTG GAG AGA CCG	Larsén and Rydin [2]
	rbcL-885R	GAT CCC CAC CGG ACA TAC GCA ATG C	Larsén and Rydin [2]
	rbcL-1409R	TCA AAT TCA AAC TTG ATT TCT TTC CA	Wikström and Kenrick [3]
rpoC1			
(part 1)	rpoC1-1-150F	GAA CCT TGT CGC TCC GCA TCC	The present study
	rpoC1-1-1073R	GAT GGA GAT CAA ATG GCT GTC C	The present study
	rpoC1-1-2113R	CCA AGC CTA TAA CTA ACA AAC CTA C	The present study
(part 2)	rpoC1-2-1385F	GGT GTA ATG AAA GAG AGG GAC C	The present study
	rpoC1-2-1971F	GGT AAC ATC TCC CCC AGT AG	The present study
	rpoC1-2-2509R	CCA TCG GAG TAG AGC GAG AAC C	The present study
(part 3)	rpoC1-3-2509F	GGT TCT CGC TCT ACT CCG	The present study
	rpoC1-3-468F	CCG CAC ATG CTG ATT CGA TG	The present study
	rpoC1-3-485F	TAC CGC ACA TGC TGA TTC GA	The present study
	rpoC1-3-485R	TCG AAT CAG CAT GTG CGG TA	The present study
	rpoC1-3-1006R	ATG CCG AAA CCC GTC AGA AA	The present study
	rpoC1-3-3919R	GAA AGA TAC GAA CGG GAA GC	The present study
trnV^{UAC}			
	trnV-365F	CCA GGA GAA CAG TAG CAT GAC	The present study
	trnV-189F	CCA CCT TGA CAG AAG TTA ATA G	The present study
	trnV-703R	GCC ATC CTT TCT TCT TCC	The present study
	trnV-703F	GGA AGA AGA AAG GAT GGC	The present study
	trnV-1656R	GCT GAA TGG AAT GAA CAA C	The present study
ycf1			
(part 1)	ycf1-1-36F	GGA TAA AGC AGC TAT TAA TAA CC	The present study
	ycf1-1-390F	CCT TCT CAT TTG AAG TGG CGT G	The present study
	ycf1-1-1044F	GTG TGA GTG ATG GGA GAC AAA G	The present study
	ycf1-1-782R	GAT ACG GGA AGT TCG AGC	The present study
	ycf1-1-1367R	CAR GGK GAT TTT GGT ATT GC	The present study
(part 2)	ycf1-2-782F	GCT CGA ACT TCC CGT ATC	The present study
	Ycf1-2-1171F	GAA GGA ATG GAT TGA TAC CG	The present study
	ycf1-2-1367F	GCA ATA CCA AAA TCM CCY TG	The present study
	ycf1-2-1741F	CTA CGT TCT CTT CTC CGG GC	The present study
	ycf1-2-2266R	CCA GGA TCA CGC CAC TTC	The present study
	ycf1-2-2765R	CAA TCT GAT TAC TTT CCG GGG C	The present study
	ycf1-2-2926R	CAT ACT TCT TCT RTG AAA	The present study
(part 3)	ycf1-3-2135F	GAT TGG AAT GAT TGG AAT AAR G	The present study
	ycf1-3-3033R	CCC ATT HTT AAT TGT ATD TTG AAC C	The present study
(part 4)	ycf1-4-2926F	GAA TTT TCA YAG AAG AAG TAT G	The present study
	ycf1-4-3297R	TCT CTT CCA AAT CCC GTG GC	The present study
	ycf1-4-3903R	CGT GTA TRA AAC TAA AGT CAC	The present study
(part 5)	ycf1-5-3761F	GTC AAT RRT AGA AAG AAK ART G	The present study
	ycf1-5-4661R	CAA ATT YTT RAC ATA GCG AAT CG	The present study
ycf66			
	ycf66-515F	GGA TCT AAA CGC CAA CCC TG	The present study
	ycf66-alt2F	CAC ATT TTC TCT CCC ACC TG	The present study
	ycf66-485F	CTT AGA AGC ATT TGG GAT AAT AG	The present study

ycf66-848R	GGA GTA TAY CAT GAG CCC AAT G	The present study
ycf66-1503R	CTG CAC TGT TCA TTC TYA TTC	The present study
ycf66-1504R	TCC CAC TGC CTT CTC ACC TA	The present study

Primers in grey are “internal” or alternative primers. Numbers included in the primer name denote approximate position within the gene region; F= forward primer; R=reverse primer.

References

1. Cheng T, Xu C, Lei L, Li C, Zhang Y, Zhou S. Barcoding the kingdom Plantae: new PCR primers for ITS regions of plants with improved universality and specificity. Mol Ecol Resour. 2016;16:138-49.
2. Larsén E, Rydin C. Disentangling the phylogeny of *Isoetes* (Isoetales), using nuclear and plastid data. Int J Plant Sci. 2016;177(2):157-74.
3. Wikström N, Kenrick P. Phylogeny of Lycopodiaceae (Lycopsida) and the relationship of *Phylloglossum drumondii* Kunze based on *rbcL* sequence data. Int J Plant Sci. 1997;158:862-71.
